# Identification of a Novel Linker Enabling the Bioconjugation of a Cyclic Dinucleotide for the STING Antibody-Drug Conjugate TAK-500

**DOI:** 10.1021/acs.bioconjchem.5c00424

**Published:** 2025-10-27

**Authors:** Hong Myung Lee, Kojo Abdul-Hadi, Vicky A. Appleman, David Cardin, Linlin Dong, Dylan England, Michelle L. Ganno, Rachel Gershman, Kenneth Gigstad, Nanda Gulavita, Zhigen Hu, Jian Huang, Shih-Chung Huang, David Lok, Liting Ma, Jenna Malley, Miho Mizutani, Nina Molchanova, Konstantin I. Piatkov, Elise Rice, Zhan Shi, Stepan Vyskocil, Jianing Wang, He Xu, Tianlin Xu, Dong Mei Zhang, Ji Zhang, Adnan O. Abu-Yousif

**Affiliations:** Takeda Pharmaceuticals International Company, Cambridge, Massachusetts 02139, United States

## Abstract

STING activates the innate immune system by inducing type-1 interferon (IFN) production and has been pursued as a therapeutic option in immuno-oncology. The targeted delivery of STING agonists to CCR2+ immune cells could enhance the therapeutic window of the agonists by selectively activating the STING pathway within targeted immune cells. The chemistry strategy was established to enable the targeted delivery of the cyclic dinucleotide STING agonist dazostinag to CCR2+ cells through an antibody-drug conjugate (ADC) approach. A self-immolative spacer between the adenine of dazostinag and the Cathepsin-B cleavable Val-Ala dipeptide linker rendered a linker payload that exhibits strong plasma stability while allowing the rapid payload release upon internalization into lysosomes. The stochastic cysteine conjugation of the dazostinag containing these linkers provided ADC TAK-500 and its mouse surrogate mTAK-500 with DAR = 4. In syngeneic tumor-bearing mouse models, mTAK-500 showed target specific antitumor activity as well as the induction of immune-stimulating cytokines.

## Graphical abstract

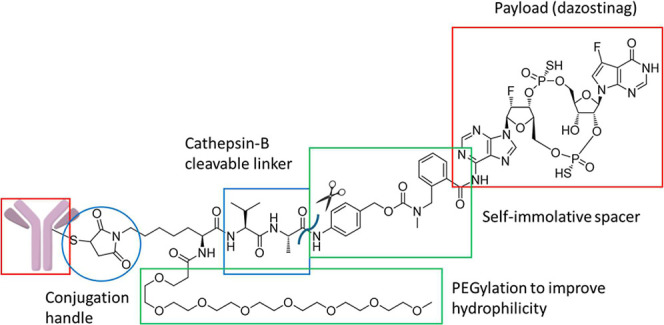

## Introduction

The success of novel immuno-oncology therapies, including PD-1 and CTLA-4 immune checkpoint inhibitors as the most notable examples, has transformed oncology research. Despite recent progress in immuno-oncology, many oncology patients continue to be nonresponsive to immune-modulating therapies due to the lack of recruitment of immune cells into the tumor microenvironment (TME). The conversion of poorly immune-infiltrated tumors, or “cold” tumors, into immune infiltrated “hot” tumors, has been intensely pursued to improve the effectiveness of the immunotherapies.^1^

Stimulator of interferon genes (STING) is an endoplasmic reticulum signaling protein that is activated by cyclic GMP–AMP (cGAMP), a cyclic dinucleotide (CDN) produced by the cyclic GMP–AMP synthase (cGAS) when it detects cytosolic DNA. The activation of the STING pathway leads to the induction of type-I interferons (INFs) and proinflammatory cytokines resulting in the enhancement of innate and adaptive immunity.^2^ Dazostinag (TAK-676) (Figure 1a) is an intravenously administered cyclic dinucleotide STING agonist that has exhibited antitumor activity driven by IFN production and immune cell activation in preclinical experiments^3^ and was investigated in a phase I clinical trial as an anticancer therapeutic (clinicaltrials.gov ID NCT04420884).

**Figure 1:**
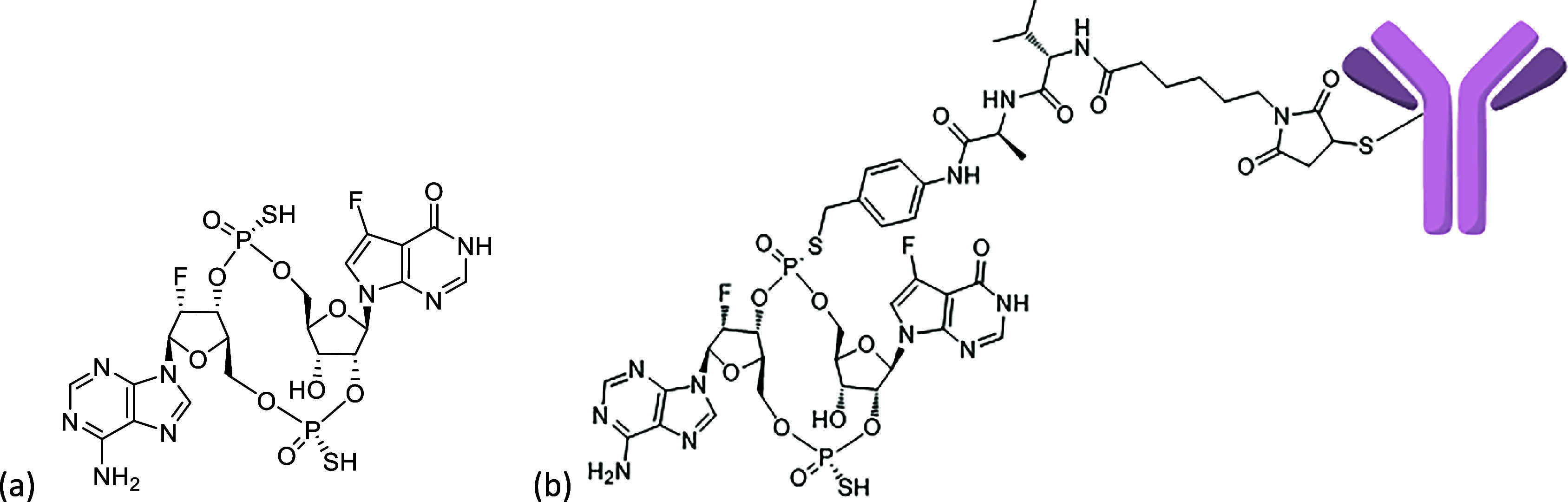
(a) Structure of STING agonist dazostinag and (b) structure of ADC linked via the sulfur atom of dazostinag.

Potential risks of systemic administration of STING agonists include activating multiple immune cell types within nontumor tissues. Targeted delivery via an antibody-drug conjugate (ADC) approach could enhance the therapeutic window of STING agonists by reducing undesired systemic proinflammatory responses, inducing the accumulation of the agonists within the targeted intratumor immune cell population, and extending the half-lives of the conjugated small molecules. Immune cells, being highly responsive to STING agonists and the key drivers of STING driven efficacy, are ideal targets for targeted delivery. Chemokine receptor 2 (CCR2) is a cell surface receptor for chemokine ligand 2 (CCL2) and regulates the migration of monocytes to sites of inflammation, in addition to being expressed by immune cells including T cells and innate myeloid cells.^4^ While CCR2+ cells adopt an immunostimulatory phenotype in the presence of the proinflammatory signal, they can also differentiate into immune-suppressive cell types under an immune suppressive environment frequently found within the TME.^5^ The evaluation of the CCR2 expression across tumor tissues revealed an increased CCR2 expression in tumor-resident immune cells in multiple indications, including non-small cell lung cancer (NSCLC).^6^ These findings support the strategy of targeted delivery of an STING agonist toward CCR2+ immune cells aiming to drive the induction of innate and adaptive immune responses.

The rapid expansion of the ADC field continues with an increasing number of molecules progressing into clinical trials. While most ADCs at clinical stages carry cytotoxic compounds as conjugated small-molecule drugs, often referred to as “payloads”, recently small-molecule drugs with a distinct mechanism of action have been increasingly adopted as ADC payloads, including immune modulating molecules targeting STING,^7,8^ TLR,^9,10^ and glucocorticoid receptor modulators (GRMs).^11,12^ We describe our chemistry strategy to conjugate the CDN STING agonist dazostinag to a CCR2-targeting antibody (TAK-202),^13^ resulting in an immune cell-directed antibody drug conjugate (iADC) TAK-500, enabling the targeted delivery of dazostinag to CCR2+ immune cells.

## Results and Discussion

### Linker Optimization

For the targeted delivery of dazostinag by the ADC approach, the linkers connecting an antibody and a small-molecule drug must satisfy two criteria: (1) the linker needs to have acceptable plasma stability, as a premature linker cleavage and payload release in the plasma would lead to systemic exposure, which in turn could result in the reduced efficacy and off-target toxicity. (2) Once the ADC is internalized into the target cells and trafficked to lysosomes, the linker needs to be rapidly cleaved for payload release, as a noncleavable linker attached to dazostinag would lead to a significant potency loss as an agonist. Cathepsin B-cleavable dipeptide linkers, including Val-Ala, have been shown to meet both criteria and are widely used in the field.^14,15^

The unique structures of CDNs, including dazostinag, present significant challenges in using them as ADC payloads. The syntheses of CDNs and their analogs are difficult to optimize as even subtle deviations from the optimal processes often result in substantially decreased yields with byproducts challenging to remove. In addition, dazostinag does not contain the functional groups, including amines, anilines, and phenols, that have widely been used as handles for ADC linkers. The use of the functional groups constituting dazostinag for ADCs was unprecedented; therefore, each functional group needed exploration as a potential ADC linker handle.

We started by exploring the phosphorothioate group of dazostinag as a handle for the ADC linkers. A cleavable linker mc (maleimidocaproic acid)-Val-Ala-*p*-aminobenzyl (PAB) group was directly added to one of the sulfur atoms of dazostinag (Figure 1b). The chemical stability of the resulting linker payload was unacceptable, as the construct was slowly degraded in water at 4 °C, resulting in a complete payload loss in 7 days. The deconjugation mechanism is unclear, as demonstrated by the degradation of a structurally related compound at pH 5 and 7, which resulted in multiple unidentified degradants (Table S3, Figure S3). Attempts to add a linker to the secondary hydroxyl group on the ribose ring were unsuccessful due to the synthetic challenges of activating the sterically hindered alcohol.

Next, we started experimenting with the amino group on the adenine of dazostinag. Anilines have been widely used as handles for ADC linkers with Val-Ala as a cleavable functional group upon internalization.^16,17^ While dazostinag lacks aniline, we hypothesized that the addition of the Val-Ala dipeptide to the adenine would result in a chemically stable amide bond that would be readily cleaved in the presence of Cathepsin B and other lysosomal proteases. We prepared intermediate **1** (Scheme 1a) to examine whether the linker payload has acceptable chemical stability, and the payload is rapidly released under lysosomal conditions. Intermediate **1** was shown not to meet either criterion: the amide bond was not chemically stable and spontaneously hydrolyzed to release free dazostinag, with 25% of the payload lost in 6 days in water at pH 7 and 37 °C (Table S2, Figure S2), while treating **1** with a rat lysosomal extract (tritosomal mixture) led to little payload release within 24 h.

**Scheme 1:**
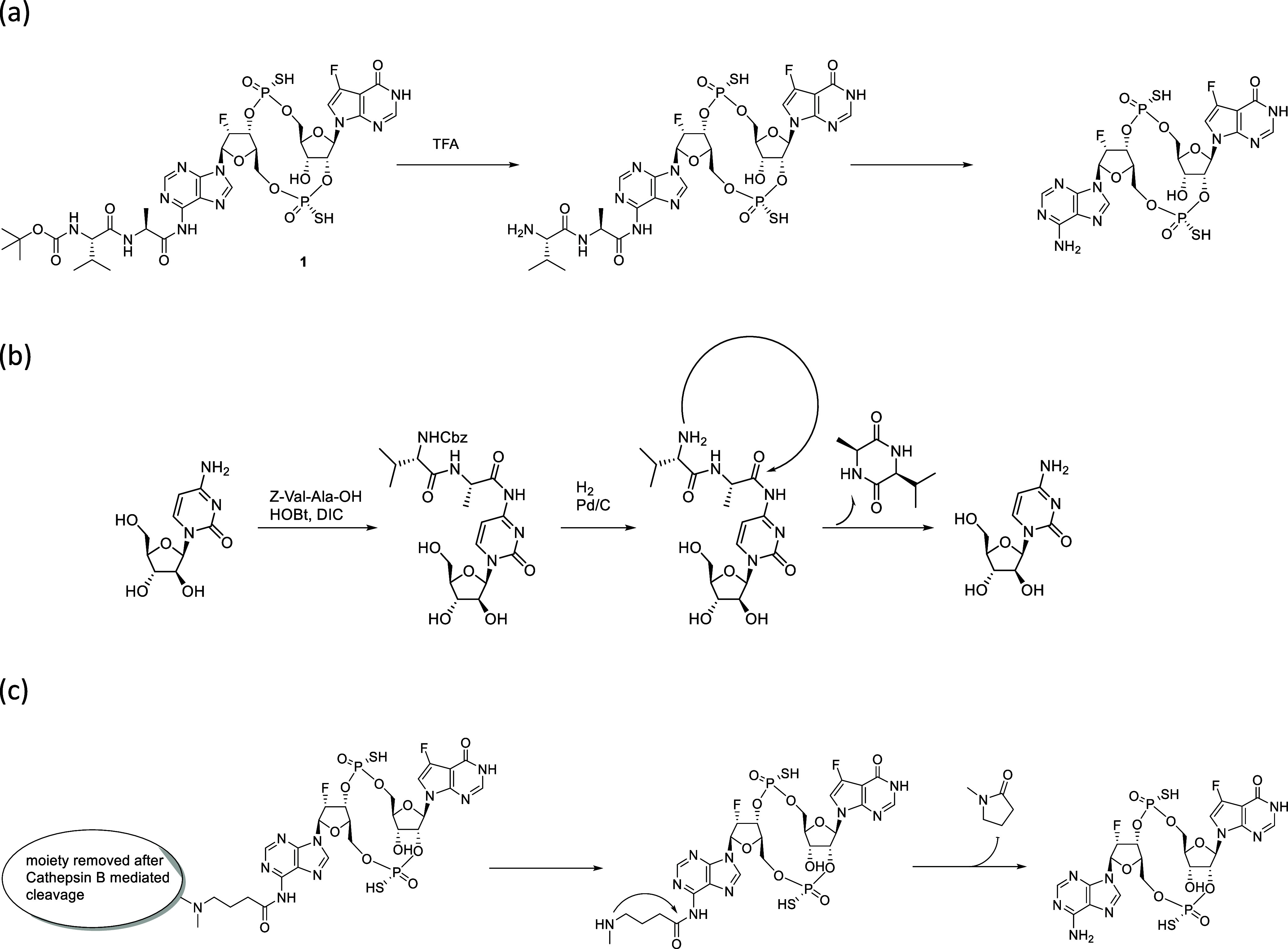
Self-Immolation of Spacers Attached to the Nitrogen on the Nucleobase. (a) Observed Self-Immolation of **1** after Boc Removal. (b) Self-Immolative Spacer Attached to Cytosine. (c) The Proposed Mechanism of Self-Immolation of a Spacer Following Cathepsin-B-Mediated Linker Cleavage

The synthetic manipulation of **1** proved to be difficult. We observed that the deprotection of Boc on the N-terminus of Val led to an immediate self-immolation of Val-Ala, yielding free dazostinag (Scheme 1a). Literature research revealed a similar observation from other researchers.^18^ In the report, the authors noticed that the removal of the amine protection group after the addition of Z-Val-Ala-OH to the amino group of cytosine led to a rapid self-immolation of the dipeptide resulting in the release of the free nucleoside (Scheme 1b). The tendency of the dipeptide appended to the amino group of nucleobases to cyclize led us to consider using a spacer linking the Cathepsin B-cleavable Val-Ala dipeptide and the adenine, which self-immolates upon the Val-Ala linker cleavage. As shown in Scheme 1c, the spacer would contain an amino group on one end and a carboxylic acid on the other, forming an amide bond between a Cathepsin B-cleavable moiety and adenine. Once the cleavable moiety is removed by the Cathepsin B-mediated linker cleavage, the amine group of the spacer is freed, triggering the cyclization of the spacer and releasing free dazostinag.

To evaluate the feasibility of such spacers, we prepared intermediate **2** (Scheme 2). This compound was chemically stable, with less than 5% degradation in water at pH 7, 37 °C for 7 days. When the compound was treated with tritosome, we observed the rapid release of dazostinag (Table S4), hypothetically via the mechanism described in Scheme 2. Intermediate **4** could be isolated but was chemically unstable and rapidly yielded free dazostinag, supporting the proposed linker cleavage mechanism.

**Scheme 2:**
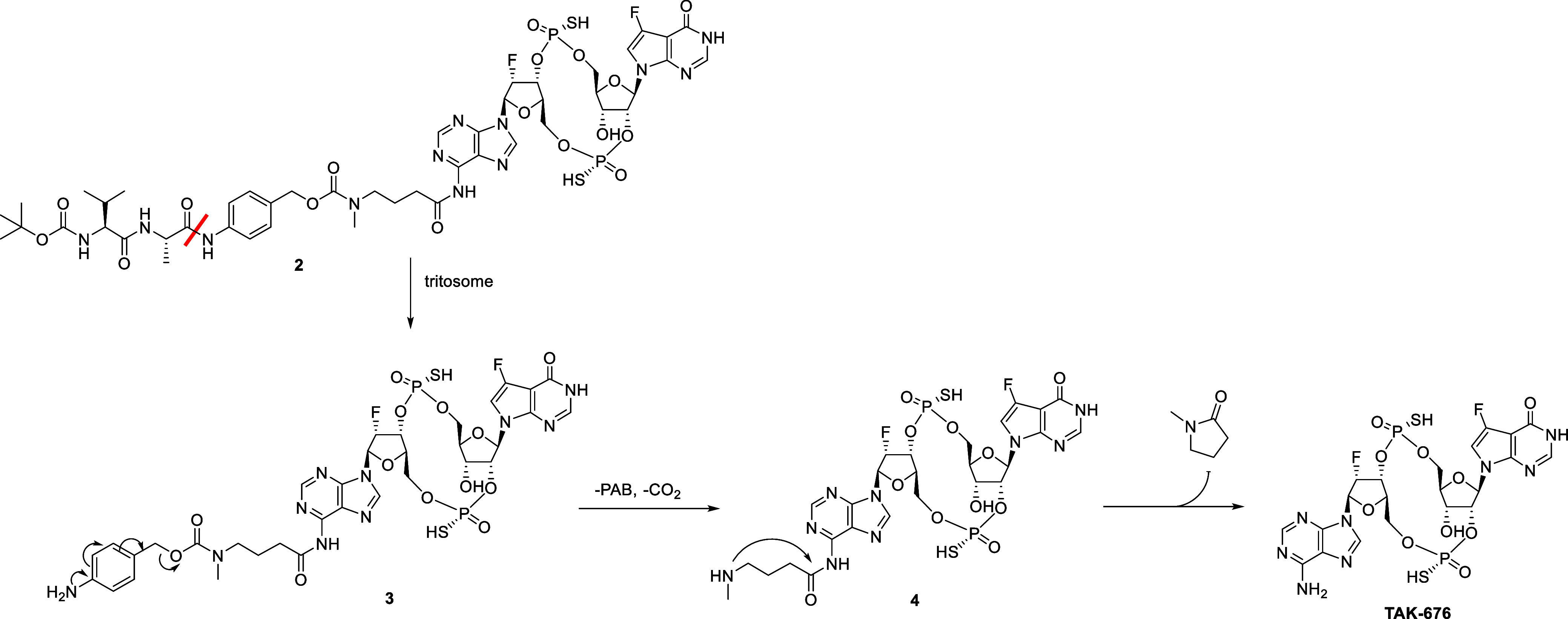
Proposed Mechanism of the Linker Cleavage from **2**

After confirming that intermediate **2** is chemically stable but rapidly cleaved upon the exposure to lysosomal extracts, we converted **2** to a full linker-payload **5** that was conjugated to TAK-202 (hIgG4 isoform) using stochastic cysteine conjugation to produce **ADC1** with an average drug-antibody ratio (DAR) of 4 (Figure 2a,b). We treated human THP-1 cells overexpressing CCR2 and a luciferase reporter gene under the control of an ISG54 minimal promoter in conjunction with five IFN-stimulated response elements with **ADC1** or an unconjugated payload dazostinag and monitored the activation of the STING pathway (Figure 2c). We observed the robust STING pathway activation after treating THP-1 cells with **ADC1** with EC50 < 1 nM, clearly demonstrating the linker cleavage and intracellular accumulation of free dazostinag upon internalization, rapid enough to overcome the lysosomal degradation of activated STING.^19^ The EC50 from the ADC is nearly 10^3^-fold lower than the EC50 with the free payload (EC50 530 nM, Table S5). The high EC50 of the free payload is attributed to the poor cell permeability of dazostinag due to its high hydrophilicity. This observation confirms that the STING pathway activation is entirely driven by intracellularly released dazostinag and not by the payload released extracellularly. The EC50 of **ADC1** was also lower than the binding affinity of dazostinag to STING (*K*_d_ = 27 nM), as measured by the TR-FRET assay.^3^ This difference may again be attributed to poor cell permeability as the released dazostinag remains trapped and accumulates intracellularly over time.

**Figure 2:**
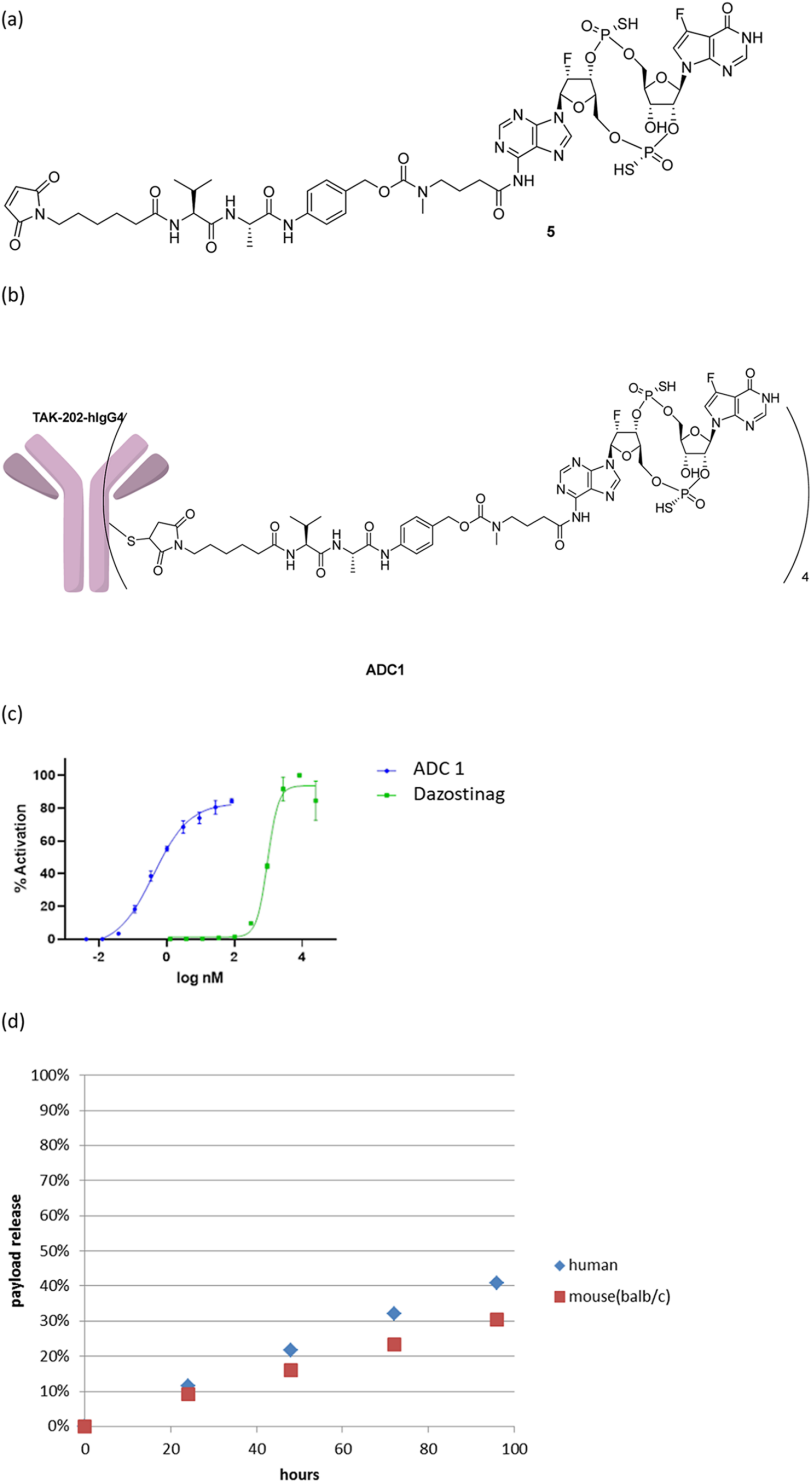
(a) The structure of linker-payload **5**. (b) The diagram of the **ADC** with linker-payload **5**. (c) In vitro STING activation treated with **ADC1** and dazostinag in CCR2-overexpressing THP-1 cells. (d) In vitro plasma stability of **ADC1** in human and mouse plasma samples.

To assess whether the linker is suitable for the in vivo use, we examined the plasma stability of **ADC1** in human and mouse samples by incubating **ADC1** in plasma samples and monitoring the amount of the released free payload over 4 days. We observed significant amounts of the released payload in day 4 from both plasma samples, 40% from the human plasma, and 30% from the mouse plasma (Figure 2d). The liability of Cathepsin B-cleavable dipeptide linkers in the mouse plasma has been well documented with carboxylesterase 1C (CES1C) identified as the main culprit.^20^ The dipeptide linkers are known to be more stable in the human plasma devoid of CES1C; therefore, the apparent premature linker cleavage of **ADC1** in the human plasma was unexpected. This led us to hypothesize that the cleavage in the plasma does not take place between Ala and *para*-aminobenzyl, as mediated by Cathepsin B or CES1C. Another potential cleavage site is the amide bond between the spacer and the amino group of the adenine. Therefore, making the amide bond between the spacer and dazostinag less susceptible to hydrolysis mediated by plasma enzymes was expected to enhance plasma stability.

With this goal, we modified the structure of the spacer as shown in Scheme 3. We added a phenyl ring adjacent to the amide bond (Scheme 3, highlighted in blue) aiming to make the amide less prone to enzyme-mediated hydrolysis.^21^ An additional benefit of this approach is the enhanced payload release due to the conformational restriction imposed by the phenyl group, positioning the methylamine in proximity to the amide, facilitating the cyclization and payload release scheme (Scheme 3). The rapid release of the free payload was observed when **ADC2**, prepared by conjugating linker-payload **6** with the modified spacer to the antibody TAK-202, was treated with a tritosomal mixture. Approximately 70% of the conjugated payload was released within 5 h, with a nearly complete payload release observed at 24 h (Table S4), which is consistent with the proposed mechanism in Scheme 3. Unlike **4**, intermediate **8** with the modified spacer could not be isolated or observed by LCMS monitoring, supporting the hypothesis that the self-immolation of the spacer is accelerated with the addition of a phenyl ring to the spacer. The addition of the phenyl ring increased the hydrophobicity of the linker payload and the resulting ADC, leading to lower yields of bioconjugations. To counter the increased hydrophobicity, a PEG chain was appended to the linker close to the maleimide, as in linker payload **9**, and stochastic cysteine conjugation of **9** to TAK-202 (hIgG1 isoform with a LAGA mutation in Fc) with average DAR = 4 provided TAK-500 (Figure 3a).^22^

**Scheme 3:**
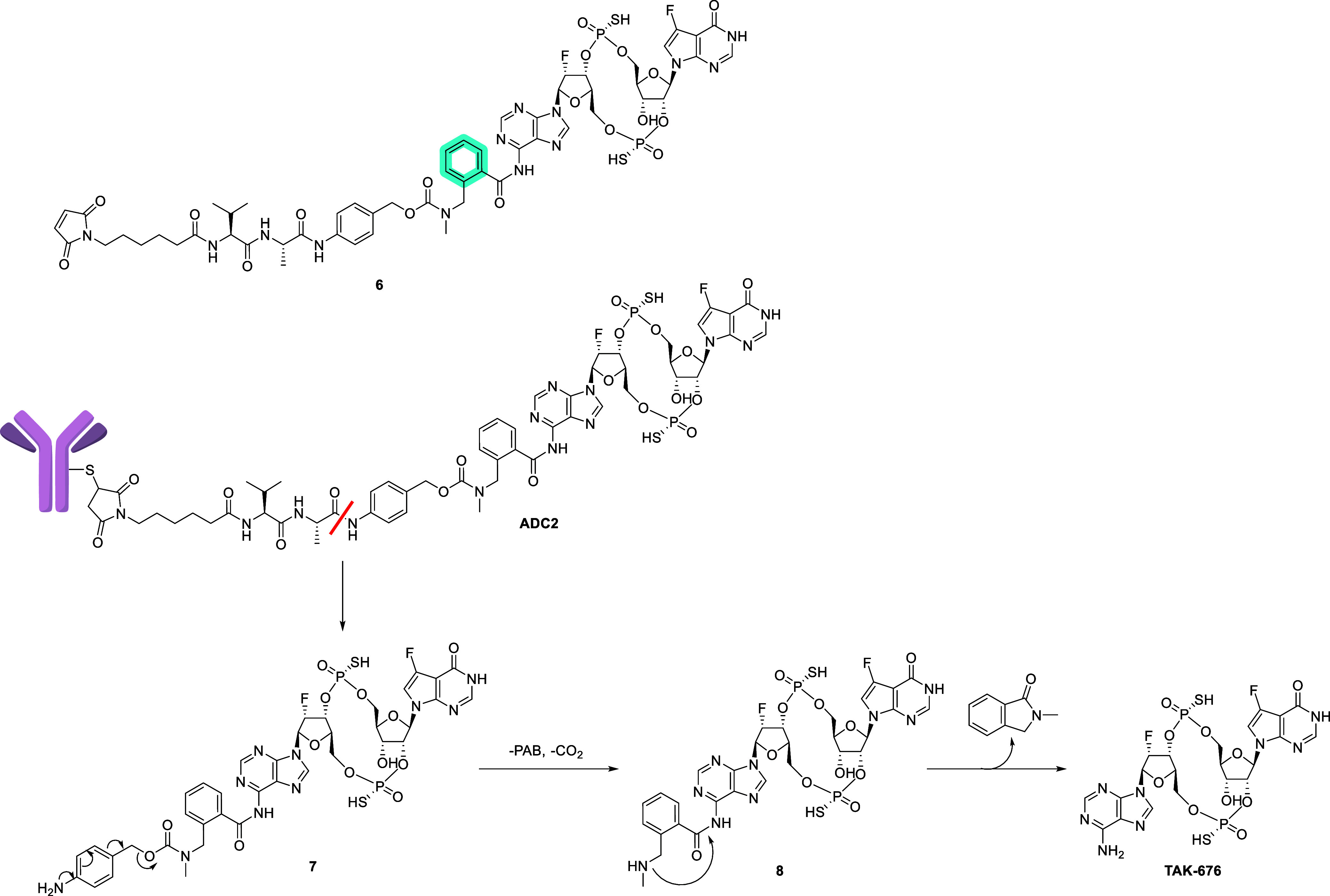
Structure of Linker Payload **6** and the Proposed Mechanism of Payload Release Following Cathepsin-B-Mediated Linker Cleavage

**Figure 3:**
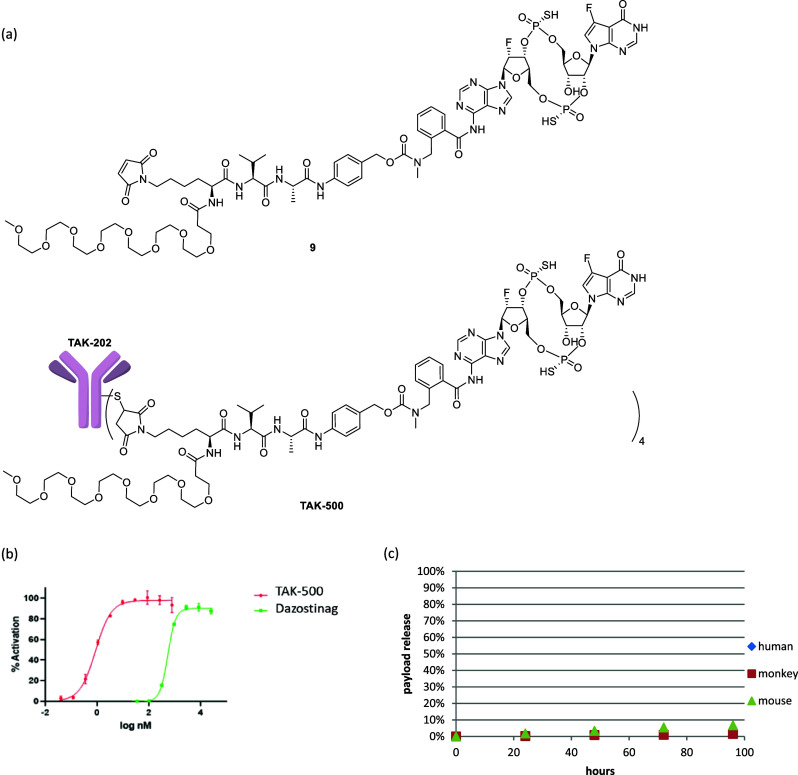
(a) The structures of linker payload 9 and TAK-500. (b) In vitro STING activation treated with TAK-500 and dazostinag in CCR2-overexpressing THP-1 cells. (c) In vitro plasma stability of TAK-500 in human, cynomolgus monkey, and mouse plasma samples.

As with **ADC1**, TAK-500 activated the STING pathway of CCR2 overexpressing THP1 reporter cells with EC50 < 1 nM, a significant potency shift compared to unconjugated dazostinag (Figure 3b, Table S5), again demonstrating the rapid payload release upon internalization. In the plasma stability evaluation, in contrast to **ADC1**, less than 10% of the payload release was observed when TAK-500 was incubated in plasma samples from the mouse, cynomolgus monkey, and human for 4 days (Figure 3c). These results confirmed that linker-payload **9** and TAK-500 meet both criteria for an in vivo use: the linker is stable in the plasma and is cleaved rapidly upon the internalization for the payload release.

The linker strategy with dazostinag was successfully applied to an alternative CDN **10**, recently reported by our group, containing a guanine rather than adenine (Figure 4).^23^ The same spacer linked Val-Ala-PABC to the amino group of the guanine of **10** via an amide bond, and the resulting linker-payload **11** was conjugated to the antibody TAK-202 to provide **ADC3** (Figure 4). The evaluation of **ADC3** in CCR2-overexpressing THP-1 reporter cells for cell activity and human/mouse/cynomolgus monkey plasma samples for plasma stability showed a very similar profile to TAK-500 (Tables S5 and S6). These results demonstrated that the same chemistry strategy can be applied to both guanine and adenine containing CDNs with both yielding linkers that have an acceptable plasma stability and rapid intracellular cleavage rate.

**Figure 4:**
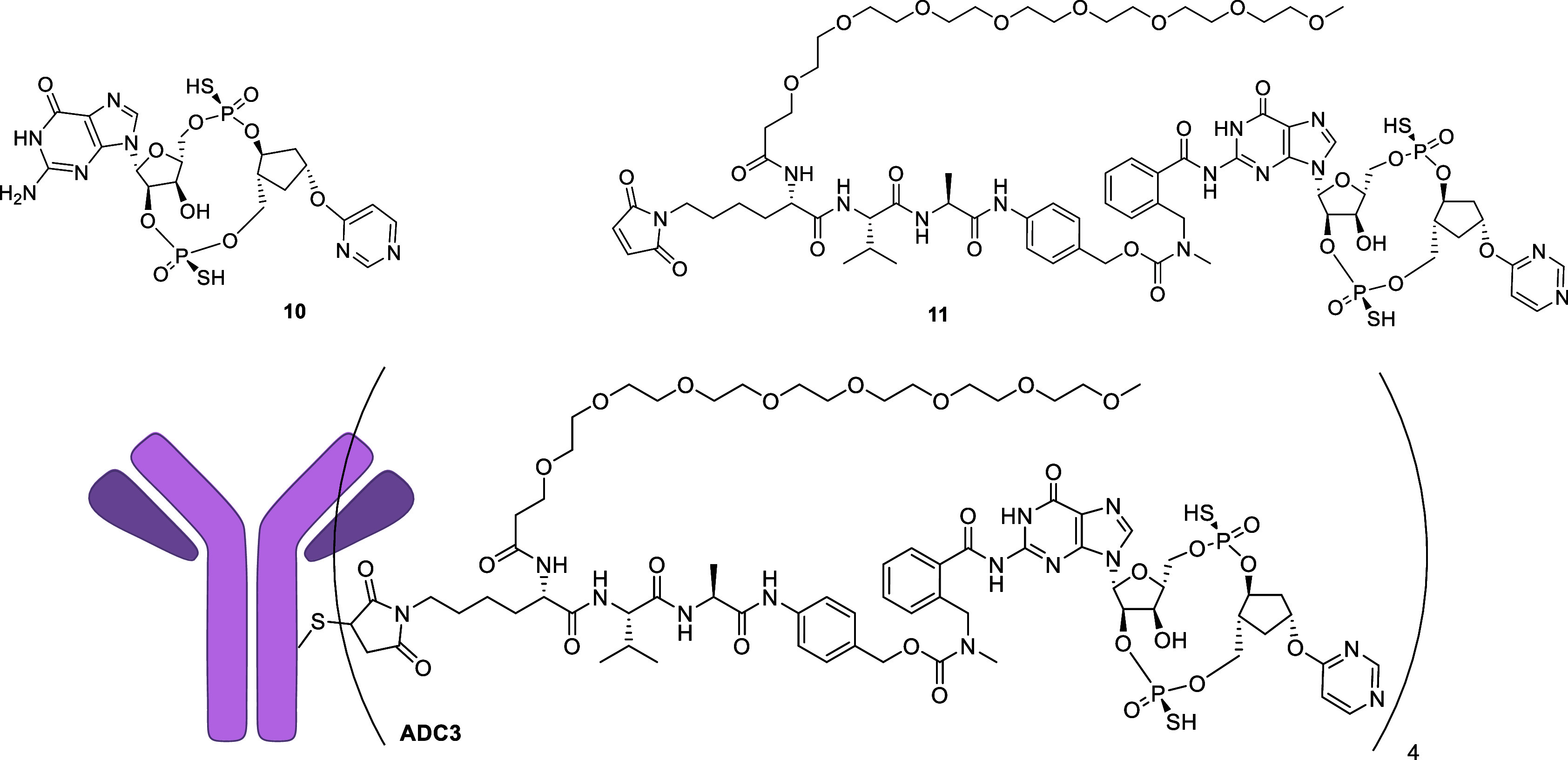
Structures of **10**, **11**, and **ADC3**.

### TAK-500 Shows an Acceptable PK Profile in Mouse and Nonhuman Primate

The plasma pharmacokinetics (PK) of TAK-500 was evaluated in mice and cynomolgus monkeys. The ADC was isolated from plasma samples by immunoprecipitation, and the concentrations of the total antibody and conjugated/unconjugated payload were measured following an established bioanalysis protocol.^24^ TAK-500 exhibited a long half-life in the plasma for both antibody (40–60 h) and the conjugated payload (17–36 h) in mice and non-human primates (NHPs) (Figure 5, Table S7). The PK parameters of the antibody from TAK-500 in the plasma were nearly identical to the parameters from the unconjugated antibody TAK-202 in NHPs (Table 1). We observed a more rapid decline of the conjugated payload concentration relative to the antibody concentration with in vivo DAR, calculated from the conjugated payload concentration divided by the antibody concentration in the plasma, declining from 4 to close to 1 in 7 days. The rates of in vivo DAR decline between mice and NHPs were similar (*k*_cleav_ was 0.016 h^–1^ for mice and 0.022 h^–1^ for NHPs). While the exposure of the conjugated payload was deemed sufficient for sustained in vivo PD and efficacy, the rate of the payload loss was greater than predicted from in vitro plasma stability assays (10% payload loss over 4 days), revealing an in vitro–in vivo disconnect.^25^ In NHPs, free dazostinag above quantifiable levels in the plasma was only observed at the first time point (1 h) from the 3 mg/kg dosing group, demonstrating that the linker is stable enough to minimize off-target toxicity caused by prematurely released payload in the serum.

**Figure 5:**
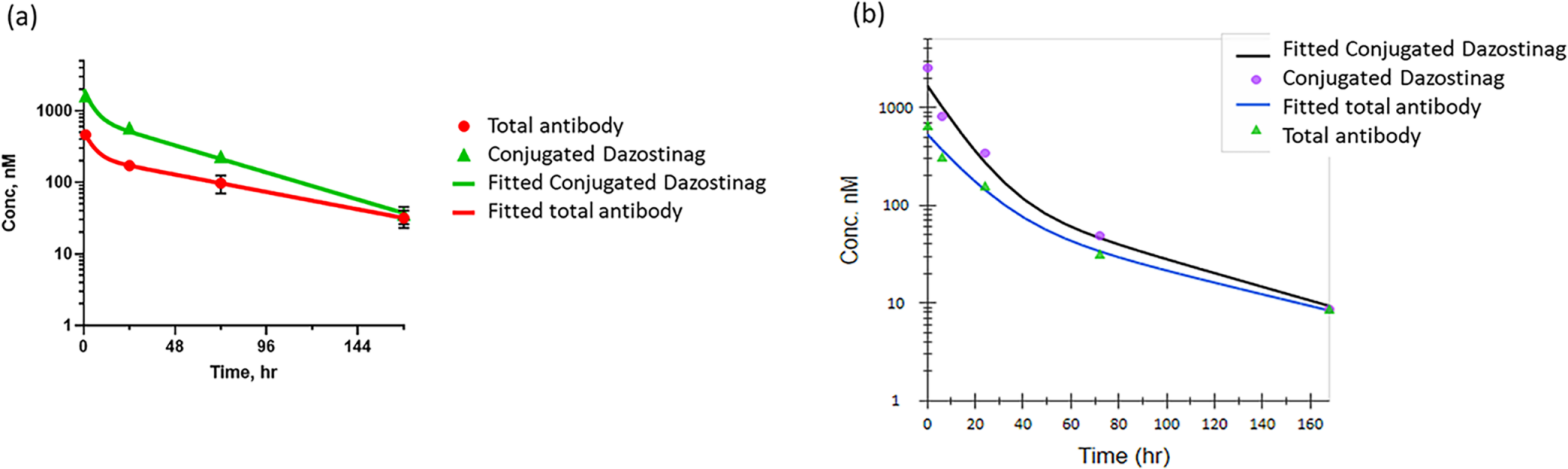
Pharmacokinetic profile of TAK-500 in (a) mouse and (b) NHP.

**Table 1:** Total Antibody Plasma Concentration in NHP after the IV Administration of TAK-500 and Its Unconjugated Antibody (TAK-202)

	TAK-202	TAK-202	TAK-500	TAK-500
antibody dose(mg/kg)	1	3	1	3
CL (mL/h/kg)	1.9 ± 0.73	0.53 ± 0.12	1.7 ± 0.4	1.4 ± 0.2
*V*_ss_ (mL/kg)	81 ± 33	39 ± 13	63 ± 19	49 ± 4
*T*_1/2_ (h)	20 ± 7	48 ± 19	52 ± 27	40 ± 11

### Isolated DAR Species of TAK-500 Exhibit a Similar PK Profile

TAK-500, prepared by stochastic conjugation on reduced interchain disulfide bonds, results in a heterogeneous mixture of DAR species. It has been well documented that higher DAR species exhibit a rapid clearance in the plasma^26,27^ correlating with their increased hydrophobicity.^28,29^ To understand how each DAR species contributes to the overall PK profile, we purified DAR 2, 4, and 6 species from TAK-500 using the preparative hydrophobic interaction chromatography (HIC), and prepared DAR 8 species separately, for PK evaluation in mice at 0.05 mg/kg (dazostinag dose). The PK analysis showed that the plasma AUC and clearance of the conjugated payload are comparable between DAR 2, 4, and 6 species (Figure 6b–e, Table S8), despite their hydrophobicity observed from analytical HIC (Figure 6a). We observed that with DAR 8 species both the antibody and the conjugated payload were more rapidly cleared than lower DAR species, but DAR 8 constitutes less than 5% of TAK-500 and, therefore, was not deemed to significantly impact the overall PK profile. In summary, the higher DAR species of TAK-500 do not pose liabilities regarding plasma PK, contrasting with the examples in the ADC literature.^26,27^

**Figure 6:**
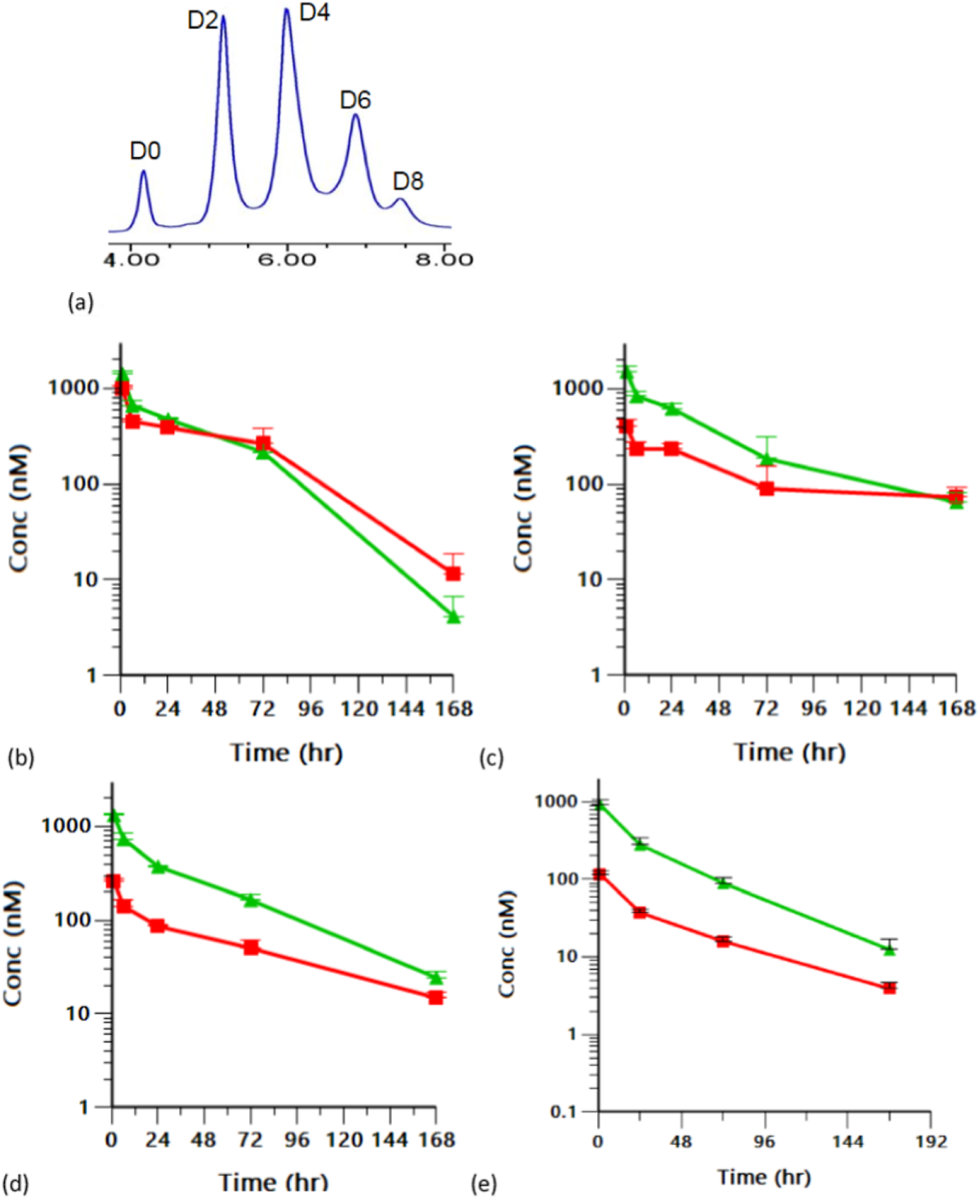
(a) HIC chromatogram of TAK-500. (b–e) Pharmacokinetic profile of the isolated DAR species TAK-500 in naïve mice, with DAR = 2, 4, 6, and 8, respectively. Green: plasma concentration of total antibody, red: plasma concentration of conjugated payload.

### Blocking Retro-Michael Type Maleimide Dissociation Does Not Improve the Exposure of the Conjugated Payload In Vivo

TAK-500 was prepared by the addition of maleimides to active thiols of cysteine. While this is one of the most common bioconjugation methods, the reaction is reversible and the maleimide slowly dissociates from the cysteine under physiological conditions (retro-Michael type dissociation).^30,31^ The spontaneous dissociation of maleimide-containing linker-payload poses the risk of deconjugation of the payloads over time and the linker-payload transfer from the ADCs to Cys-containing serum albumin.^32^ Efforts to prevent the linker-payload dissociation, and the potential risks of deconjugation, reduced efficacy, and off-target toxicities, include hydrolyzing the succinimide after the addition of the thiol to the maleimide^33,34^ and using nonmaleimide electrophiles such as bromoacetamides.^11,30^

To investigate whether blocking the spontaneous dissociation leads to a more desirable PK profile with a slower deconjugation rate, the linker-payload **9** was modified to contain a maleimido-acetyl group (linker-payload **12**, Figure 7a) that has been reported to undergo hydrolysis following the thiol addition to the maleimide under mild conditions to produce an adduct that does not spontaneously dissociate.^35^ The resulting ADC from the conjugation of **12** to TAK-202 was incubated in pH 8 buffer for 24 h, and the complete hydrolysis of the succinimide was observed by MS analysis (**ADC4**, Figure 7b, succinimide highlighted in blue). **ADC4** was stable even in the presence of an excess amount of glutathione (2 mM for 10 μM of ADC), which would work as an electrophile scavenger and facilitate the maleimide dissociation, unlike TAK-500 that degraded substantially under the same condition (Figure 7c), demonstrating that the linker payload does not dissociate even under forcing conditions. However, the PK profile of **ADC4** in mice is nearly identical to that of TAK-500 (Figure 7d) with a very similar clearance of the conjugated drug and the deconjugation rate (Table 2). While blocking the retro-Michael dissociation of linker-payloads has been reported to provide a more desirable PK profile and enhanced efficacy,^30,33^ preventing the linker-payload dissociation from TAK-500 did not provide a clear benefit in terms of the exposure of the conjugated CDN. This unexpected outcome may be attributed to the premature linker cleavage in the plasma, which was not captured by in vitro plasma stability assays and occurred more rapidly than the retro-Michael dissociation. Alternatively, it may indicate that the degradation of the conjugated CDN potentially by serum hydrolases such as ENPP1 ^36^ while it is still attached to the antibody, rather than the payload dissociation from the antibody, is the major clearance mechanism of the conjugated payload in the STING ADCs.

**Figure 7:**
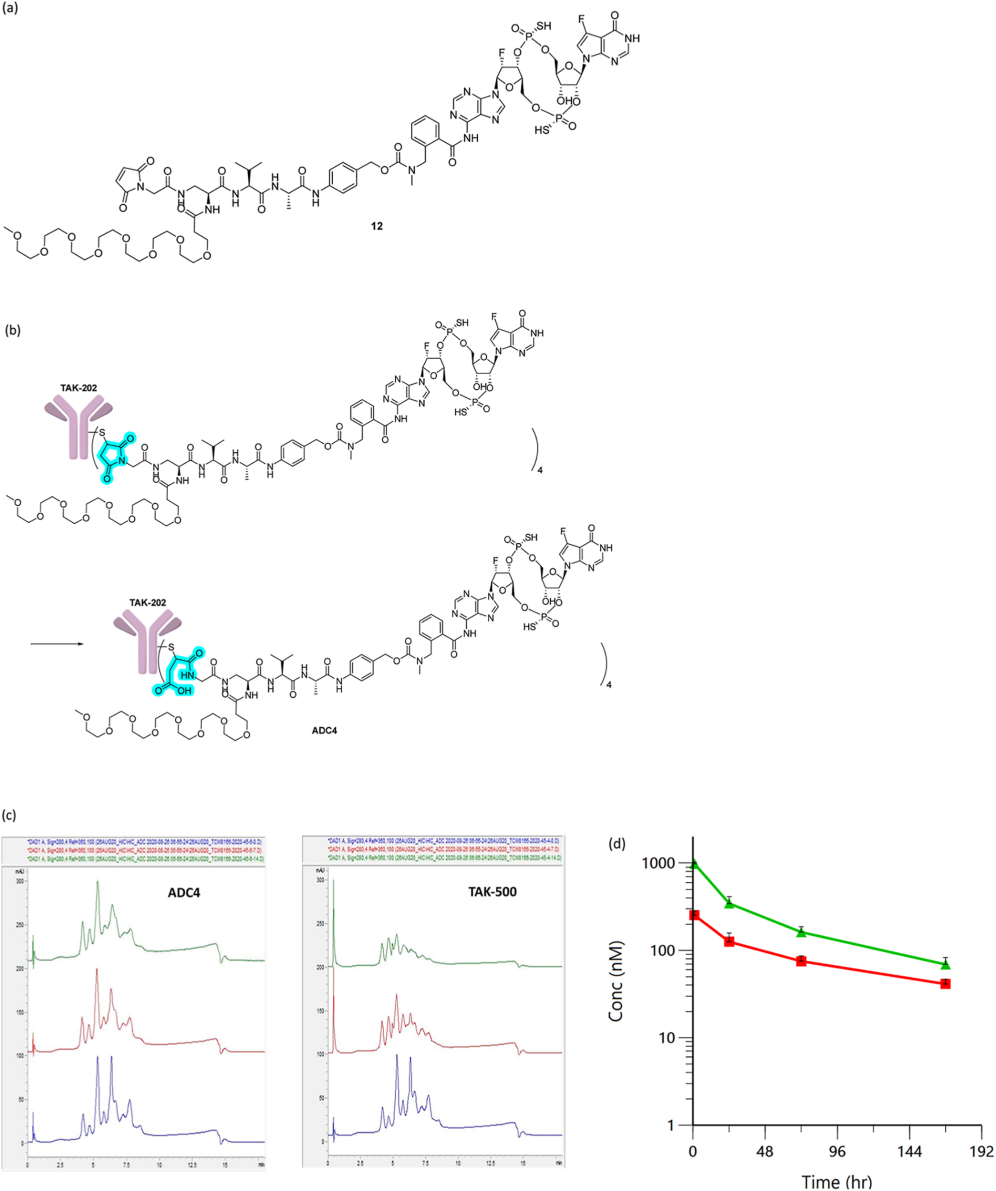
(a) The structure of linker payload 12. (b) The hydrolysis of the succinimide and the resulting structure of ADC4. (c) HIC chromatogram of **ADC4** (left) and TAK-500 (right) after the treatment with 2 mM GSH at pH 7.4, 37 °C. Blue: day 0, red: day 7, green: day 14. (d) Pharmacokinetic profile of **ADC4** in naïve mice. Green: plasma concentration of the total antibody, red: plasma concentration of the conjugated payload.

**Table 2:** Comparison of Pharmacokinetic Parameters of Total Ab and Conjugated Payload in Naive Mice after the IV Administration of **ADC4** and TAK-500 (50 μg/kg, Payload Dose)

reagent	analyte	*T*_1/2__(h)_	*C*_1h(nM)_	AUC_last(h×nM)_	AUC_INF(hr×nM)_	CL (mL/h/kg)	*V*_ss_ _(mL/kg)_
**ADC4**	total Ab	92	253	14,600	20,100	0.92	117
	conj. payload	64	983	37,100	43,500	1.6	131
TAK-500	total Ab	59	461	19,100	21,800	0.87	67
	conj. payload	36	1590	52,300	54,100	1.3	60

### Preparation of Mouse-Surrogate Antibody-Drug Conjugate mTAK-500

The antibody of TAK-500, TAK-202, does not have an affinity for murine CCR2. For pharmacology studies in mouse models, we needed a mouse surrogate ADC, mCCR2-dazostinag or mTAK-500, a conjugate of a murine-CCR2 binding antibody with a mIgG2a backbone (RtMuMC-21-S98)^37^ with linker-payload **9** via the stochastic cysteine conjugation method. The murine CCR2 antibody did not respond to the standard TAK-500 conjugation condition in the same way as TAK-202. Reduction with TCEP followed by the addition of linker-payload **9** provided a mixture with a large amount of an unconjugated antibody and DAR 7–8 species, with DAR 2–6 species smaller than expected according to HIC analysis (Figure S1a,b). We hypothesized that the reduction of the first interchain disulfide bond was rate-limiting, potentially leading to the conformational change facilitating the reduction of the other interchain disulfide bonds. To achieve the standard distribution of DAR species resembling TAK-500, we treated the murine CCR2 antibody with 20 equiv of TCEP for global interchain disulfide bond reduction, removed the excess TCEP, and then partially oxidized the disulfide bonds using dehydroascorbic acid (DHAA) before the addition of the linker payload **9**. This modified procedure provided the desired standard distribution of DAR species, confirmed by HIC and LCMS (Figure S1c).

### mTAK-500 Exhibits Acceptable PK Profile and Results in In Vivo Antitumor Activity

The PK profile of mTAK-500 was evaluated in female C57BL/6 mice bearing MC38 syngeneic tumors after a single IV administration (Table S9). In alignment with the TAK-500 mouse PK, we observed the extended exposure of the antibody and conjugated dazostinag in the plasma.

MC38 syngeneic tumor-bearing C57BL/6 mice were treated with mTAK-500 and its isotype control (prepared using the KTI-mIgG2 antibody) (single IV administration), and the tumor size was monitored. Tumor reduction was observed with the treatment of mTAK-500 at relatively low doses of 5 μg/kg, 10 μg/kg, and 25 μg/kg (dose based on the payload), with the 25 μg/kg dose causing a 10% body weight loss in mice 3 days postadministration before recovery. A greater reduction of tumor sizes from mTAK-500 compared to the isotype control at 5 and 10 μg/kg supports that the antitumor activity is target driven. To achieve significant in vivo efficacy, unconjugated dazostinag needed to be dosed at 1–2 mg/kg, >100 times higher than the required dose of mTAK-500 ^3^, which exhibits the potential of enhanced efficacy by the targeted delivery of the STING agonist. The dose-dependent increase of immune-stimulating cytokines in IP-10, CCL2 (MCP-1), IFNα, TNFα, IL-6, and IFNγ was observed following the treatment with mTAK-500 in vivo, potentially driving the observed antitumor activity. We have provided a comprehensive analysis of the in vivo data and additional preclinical investigation using mTAK-500 in a separate publication.^6^

## Conclusion

STING agonists have been pursued as immuno-oncology therapeutic options, as they have the potential to activate the innate immune response within the tumor microenvironment and overcome the resistance against immune checkpoint inhibitors. CCR2 is a cell surface receptor expressed on various immune cells, including T cells and myeloid cells. We hypothesized that the CCR2-targeted delivery of an STING agonist has the potential for an enhanced therapeutic index due to selective and enhanced STING activation within CCR2+ immune cells. Here, we described a chemistry strategy for the bioconjugation of a STING agonist dazostinag on the CCR2-targeting antibody TAK-202.

The criteria for an optimal ADC linker are (1) sufficient plasma stability to prevent premature payload loss into circulation and (2) rapid linker cleavage within the lysosome upon cellular internalization to induce a strong cellular potency. We chose a Cathepsin-B cleavable Val-Ala linker that meets both criteria and attempted to attach it to the NH_2_ group on the adenine of dazostinag. After multiple design cycles, we learned that the addition of a self-immolative spacer between the NH_2_ group and cleavable Val-Ala linker provided linker payload **9** with acceptable cellular activity and plasma stability. The stochastic cysteine conjugation of **9** to a CCR2-targeting antibody with an average DAR of 4 resulted in TAK-500.

TAK-500 exhibited an acceptable PK profile in mice and NHPs with the exposure of the conjugated payload sufficient for sustained in vivo efficacy. We observed a similar exposure of the conjugated payload from the isolated DAR 2, 4, and 6 species in the plasma in mouse PK evaluations. The modification of the maleimide to block retro-Michael type linker dissociation led to a more chemically stable ADC; however, it did not enhance the conjugated payload plasma exposure in vivo, which may indicate a disconnect between the linker stability measured in vitro and in vivo or payload metabolism before its release from the antibody. A mouse surrogate of TAK-500 was prepared by the complete reduction/partial oxidation of interchain disulfide bonds, followed by the addition of **9** to provide a statistical mixture of DAR species. Robust antitumor activity and an increase of immune-stimulating cytokines were observed when MC38 syngeneic tumor-bearing mice were treated with mTAK-500. The observed strong antitumor activity, achieved by a single administration of mTAK-500 at the dose >100-fold lower than the unconjugated dazostinag, demonstrates the advantage of targeted delivery of the STING agonist. TAK-500 was evaluated in a phase 1 clinical trial (clinicaltrials.gov ID NCT05070247).

## Supplementary Material



## Notes

Synthetic procedures for intermediates, linker payloads, and ADCs; NMR spectrum of compounds **5**, **9**, **11**, and **12**; HIC/SEC/LCMS of **ADC3**, **ADC4**, TAK-500, and mTAK-500; in vitro tritosomal, chemical, and plasma stability assay, and THP1 reporter cell assay protocols and data; mouse in vivo PK and efficacy study protocols; and mouse and NHP PK parameters with TAK-500 and mTAK-500 (PDF)
