# Supplementary material for: Identification of a Novel Linker Enabling the Bioconjugation of a Cyclic Dinucleotide for the STING Antibody-Drug Conjugate TAK-500
Source: Bioconjug Chem. 2025 Oct 27;36(11):2423–35. doi: 10.1021/acs.bioconjchem.5c00424 (PMC12635970; doi:10.1021/acs.bioconjchem.5c00424)

**Supporting Information**

# Identification of a Novel Linker Enabling Bioconjugation of a Cyclic Dinucleotide for the STING Antibody-Drug Conjugate TAK-500

Hong Myung Lee*, Kojo Abdul-Hadi, Vicky A. Appleman, David Cardin, Linlin Dong, Dylan England, Michelle L. Ganno, Rachel Gershman, Kenneth Gigstad, Nanda Gulavita, Zhigen Hu, Jian Huang, Shih-Chung Huang, David Lok, Liting Ma, Jenna Malley, Miho Mizutani, Nina Molchanova, Konstantin I. Piatkov, Elise Rice, Zhan Shi, Stepan Vyskocil, Jianing Wang, He Xu, Tianlin Xu, Dong Mei Zhang, Ji Zhang, Adnan O. Abu-Yousif.

Takeda Pharmaceuticals International Company, Cambridge, Massachusetts 02139, United States

## Table of Contents

Linker-payload synthesis ........ S1
Bioconjugation ........ S12
Safety ........ S15
Chemical stability assessment ........ S15
Tritosomal stability assay ........ S18
Plasma Stability Assay ........ S19
THP1 Dual Lucia Reporter Gene Assay ........ S19
Pharmacokinetics evaluation in naïve mice ........ S20
Assessment of mTAK-500 Pharmacokinetics in Tumor Bearing Mice ........ S21
In Vivo Assessment of mTAK-500 Efficacy in Tumor Bearing Mouse Models ........ S22
Spectral data ........ S25

## Linker-payload synthesis

NMR conditions

1H NMR spectra were acquired on a 400 MHz Bruker spectrometer unless otherwise stated. 31P NMR spectra were recorded at 162 MHz on a 400 MHz Bruker spectrometer and acquired with 1H decoupling unless otherwise stated. 19F NMR spectra were recorded at 376 MHz on a 400 MHz Bruker spectrometer unless otherwise stated.

HRMS conditions

HRMS spectra were acquired using an Agilent 1260 Infinity II Bio-inert Multisampler, Bio-inert Column compartment, Bio-inert Quaternary Pump, DAD multi-wavelength detector, and an Agilent 6545 LC-QTOF mass spectrometry instruments, with an Osaka Soda Capcell PAK C1 UG120 (5 µm, 2.0 mm ID x 35 mm length) reverse-phase column. Solvent systems for LC consisted of 0.1% formic acid in water for mobile

phase A, and 0.1% formic acid in acetonitrile for mobile phase B, or equivalent solvent system. Typically, LC elution commenced at a 95%/5% mobile phase A/B ratio. After an interval of isocratic flow, typically 0.5 min, the mobile phase was changed to 100%B over a 1.5 min linear gradient. Flow rate was 0.7 mL/min for a 5 min run.

Typically, 2 uL of a 1 mM solution in DMSO was injected into the mass spectrometer. QTOF settings: gas temperature 35 °C, drying gas flow rate = 10 L/min, nebulizer pressure 55 psig, sheath gas temperature and flow were 35 °C and 12 L/min, respectively. The following voltage settings were applied: capillary voltage 4500V, nozzle voltage 2000V, fragmentor voltage 75V.

Data was analyzed by using Agilent Mass Hunter Qualitative Analysis software, version 8.07.00 SP2. Mass error [ppm] was expressed as difference between the exact and observed mass divided by exact mass multiplied by 1x106.

LCMS conditions

LCMS spectra were recorded on a Hewlett-Packard HP1100 or Agilent 1100 Series LC system connected to a Micromass mass spectrometer using reverse phase C18 columns. Various gradients and run times were selected in order to best characterize the compounds. Mobile phases were based on ACN/water or MeOH/water gradients and contained either 0.1% formic acid (methods indicated as FA) or 10 mM ammonium acetate (methods indicated as AA). One example of a solvent gradient that was used was 100% mobile phase A (mobile phase A = 99% water + 1% ACN + 0.1% formic acid) to 100% mobile phase B (mobile phase B = 95% ACN + 5% water + 0.1% formic acid) at a flow rate of 1 mL/min for a 16.5 min run.

In some cases, LCMS spectra were recorded on an Agilent 1290 Infinity UPLC system connected to an Agilent 6130 mass spectrometer, a Waters Acquity UPLC system connected to a Waters Acquity SQ mass spectrometer, or an Agilent 1100 Series HPLC system connected to a Waters Micromass ZQ mass spectrometer using reverse phase C18 columns. Various gradients and run times were selected in order to best characterize the compounds. Mobile phases were based on ACN/water or MeOH/water gradients and contained either 0.1% formic acid (methods indicated as FA) or 10 mM ammonium acetate (methods indicated as AA). One example of a solvent gradient that was used was 95% mobile phase A (mobile phase A = 99% water + 1% ACN + 0.1% formic acid) to 100% mobile phase B (mobile phase B = 95% ACN + 5% water + 0.1% formic acid) at a flow rate of 0.5 mL/min for a 5 min run.

Preparative HPLC

Preparative HPLC separations were conducted using 18x150 mm Sunfire C-18 columns eluting with water-ACN gradients using a Gilson instrument operated by 322 pumps with the UV/visible 155 detector triggered fraction collection set to between 200 nm and 400 nm. Mass gated fraction collection is conducted on an Agilent 1100 LC/MSD instrument.

Preparative SFC

Preparative SFC was conducted using 10, 20 or 30 mm x 250 mm ChiralPak columns (typically IA, IB, IC, ID, IE and IF), 10 or 20 mm x 250 mm Phenomenex Lux Cellulose-4 or 2-ethylpyridine columns eluting with appropriate percentages of supercritical carbon dioxide and alcohol containing either 0.3% diethylamine, 0.3% TEA, 0.3% formic acid or without any acid or base additives. Isocratic conditions with

flow rates in the range of 10-100 mL/min and a column temperature of 40 °C are typical. Preparative SFC is conducted on a Jasco SFC prep purification system with UV/visible triggered fraction collection set to between 200 nm and 400 nm and back pressure regulation set to 10 MPa.

**Scheme S1**

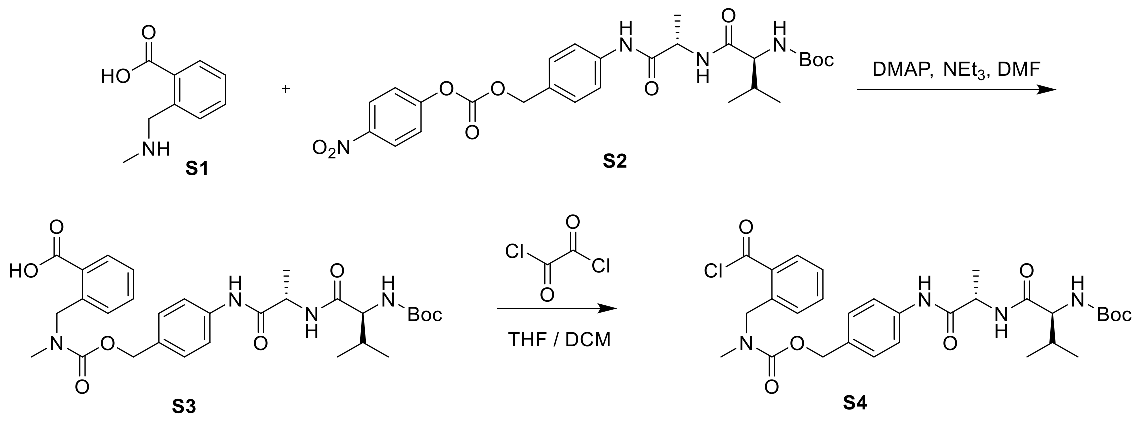


Step 1:

To a solution of tert-butyloxycarbonyl-valyl-alanyl-(4-aminobenzyl)-(4-nitrophenyl)carbonate (**S2**, 835 mg, 1.4 mmol) and 4-dimethylaminopyridine (173 mg, 1.4 mmol) in DMF (5.5 mL) and triethylamine (0.80 mL, 5.8 mmol) was added a solution of 2-[(methylamino)methyl]benzoic acid HCl (150 mg, 0.72 mmol) (**S1**, 45 mg, 0.05 mmol) in DMF (5.5 mL) at rt. The reaction mixture was allowed to stir at rt for 15 min. Following purification by silica gel chromatography (0-25% MeOH/DCM) **S3** (306 mg, 67%) was obtained. LCMS (AA): m/z = 583.4 (M-H). $^1$H NMR (400 MHz, DMSO-$d_6$) δ ppm 0.74 - 0.94 (m, 6 H), 1.31 (br s, 3 H), 1.39 (s, 9 H), 1.97 (br d, *J*=7.03 Hz, 1 H), 2.87 (s, 3 H), 2.96 (s, 2 H), 3.84 (br s, 1 H), 4.43 (br d, *J*=6.02 Hz, 1 H), 4.83 (br d, *J*=4.89 Hz, 2 H), 4.91 - 5.15 (m, 2 H), 6.74 (br d, *J*=8.91 Hz, 1 H), 7.00 - 7.22 (m, 2 H), 7.26 - 7.66 (m, 5 H), 7.80 (dd, *J*=7.65, 1.00 Hz, 1 H), 8.02 - 8.22 (m, 2 H), 9.82 - 10.21 (m, 1 H).

Step 2:

To a solution of **S3** (295 mg, 0.50 mmol) in THF (1.5 mL) cooled to 0 °C was added oxalyl chloride (2.0 M solution in DCM, 0.25 mL, 0.50 mmol) followed by 3 drops of DMF. The reaction mixture was allowed to stir at 0 °C for 45 min. The mixture was then concentrated to dryness to provide **S4** (304 mg, 100%). The product was used immediately without further purification.

**Scheme S2**

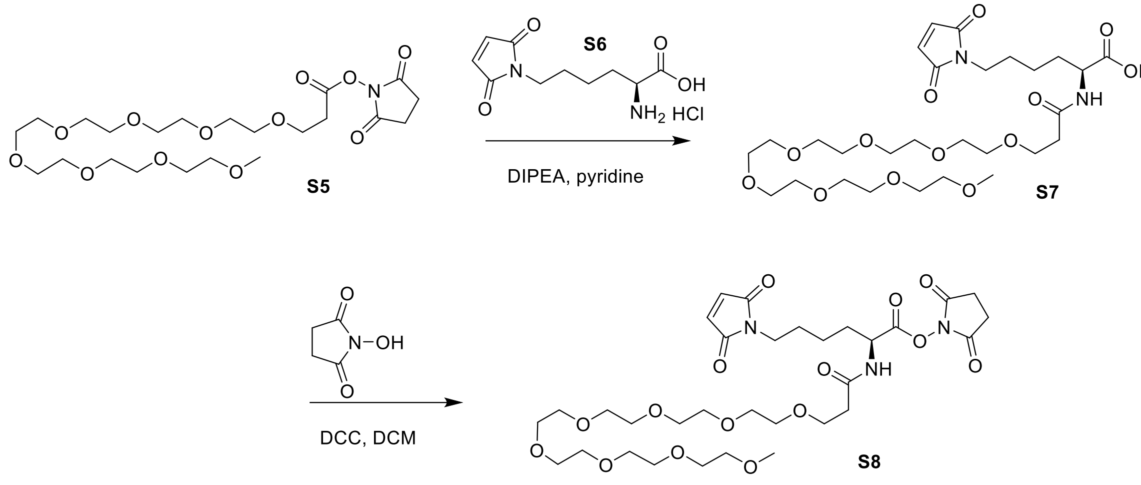


Step 1:

To a mixture of **S6** (1.05 g, 4.00 mmol) and **S5** (2.08 g, 4.04 mmol) in pyridine (21.0 mL) was added DIEA (1.43 mL, 8.20 mmol) and the mixture was allowed to stir at rt for 30 mins then cooled to 0 °C and stirred overnight. The volatile solvent was removed and the residue was azeotroped with toluene (3×20 mL) and acetonitrile (10 mL) to give **S7** (4.00 g, 100% yield) as a colorless oil. LCMS (AA): m/z = 619.3 (M-H). $^{1}$H NMR (400 MHz, DMSO-$d_6$) δ 1.24 (br d, *J*=7.83 Hz, 2 H), 1.32 - 1.35 (m, 1H), 1.47 - 1.59 (m, 1 H), 1.62 - 1.73 (m, 1 H), 2.28 (s, 2 H), 2.31 - 2.44 (m, 2 H), 2.87 (q, *J*=6.40 Hz, 2 H), 3.37 - 3.84 (m, 28 H), 4.06 - 4.20 (m, 1 H), 6.63 - 6.77 (m, 1 H), 7.16 (d, *J*=7.58 Hz, 2 H), 7.23 (d, *J*=7.34 Hz, 1 H), 8.06 (d, *J*=7.70 Hz, 1 H).

Step 2:

A mixture of N-hydroxysuccinimide (469 mg, 4.08 mmol), N,N'-dicyclohexylcarbodiimide (824 mg, 4.00 mmol), and the crude **S7** from step 1 (4.00 g, 4.00mmol) in DCM (76.7 mL, 1200 mmol) was allowed to stir at rt for 16 h. Solid crashed out from this reaction was filtered and the filtrate was concentrated to give a crude oil. Purification by silica gel chromatography (0-20% IPA/DCM) gave **S8** (1.70g, 52%) as a colorless oil. LCMS (AA): m/z = 735.3 (M+$NH_4$). 1H NMR (400 MHz, METHANOL-d4) δ 1.42 - 1.55 (m, 2 H), 1.64 (m, 2 H), 1.81 - 1.93 (m, 1 H), 1.94 - 2.01 (m, 1 H), 2.52 (m, 2 H), 2.81 - 2.86 (m, 4 H), 3.36 (s, 3 H), 3.51 - 3.57 (m, 4 H), 3.60 - 3.66 (m, 26 H), 3.68 - 3.76 (m, 2 H), 4.03 - 4.17 (m, 2 H), 4.74 - 4.80 (m, 1 H), 6.74 - 6.86 (s, 2 H).

**Scheme S3**

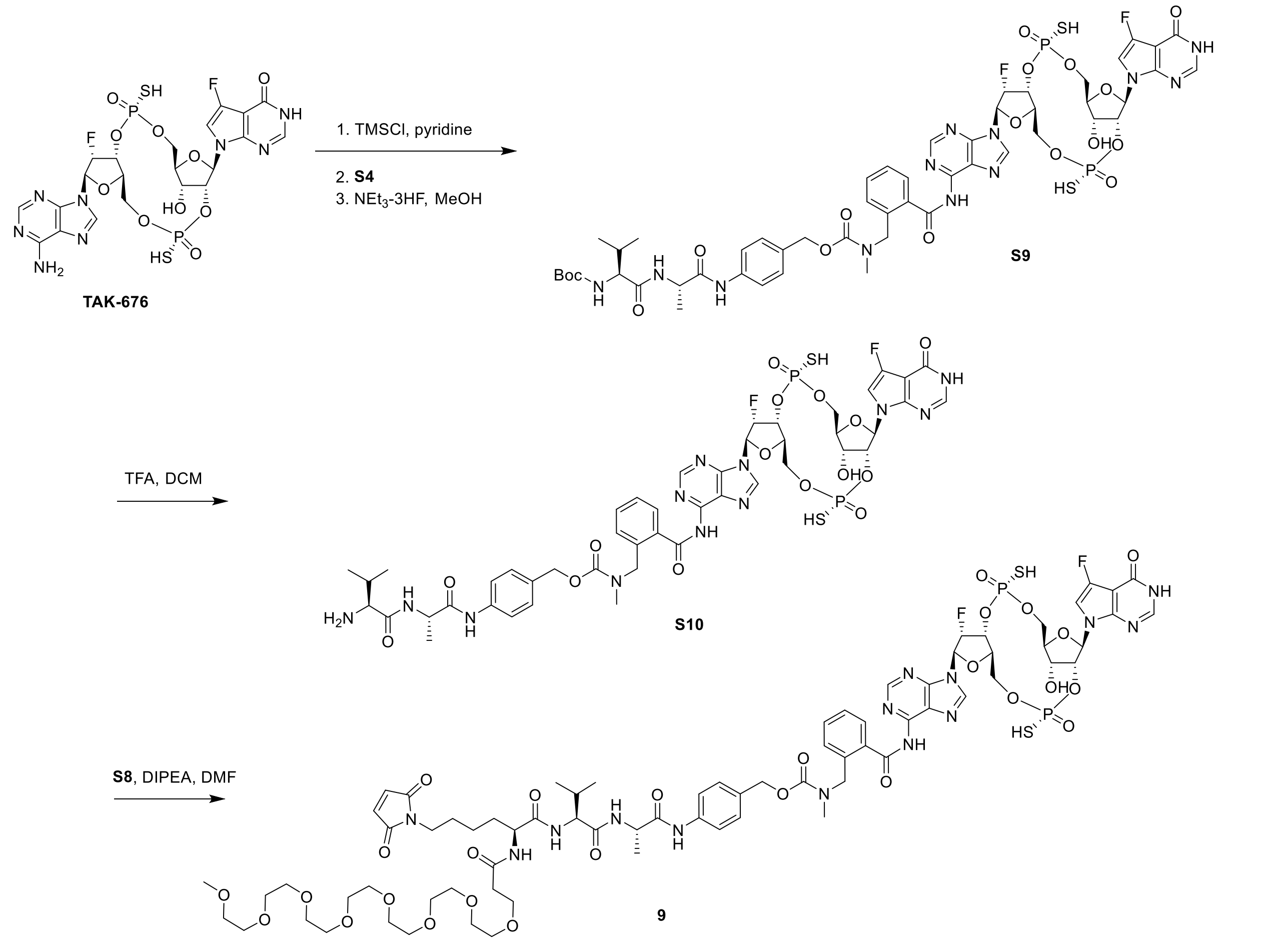


Step 1: To a 50-mL RBF was added TAK-676 (243 mg, 0.266 mmol), pyridine (3.0 mL), and TMSCl (0.276 mL, 2.13 mmol). The mixture was stirred at rt for 2h. **S4** (962 mg, 1.59 mmol) in pyridine (3.2 mL) was added to the mixture in an ice bath. After addition, the mixture was slowly warmed to rt and stirred for 12h. The reaction mixture was cooled to 0 °C and then ammonium hydroxide (20.0 mL, 598 mmol) was slowly added. The mixture was warmed to rt and stirred for 2h. The crude residue absorbed onto Celite was purified by reverse phase flash column chromatography (0-40% ACN/aqueous triethylammonium acetate (10 mM)) to provide 164mg of **S9** (0.111 mmol, 42% yield). LCMS (AA): m/z = 1277.3 (M+H).

$^{1}$H NMR (400 MHz, METHANOL-$d_4$) δ 0.84 (m, 6 H), 1.16 (m, 3 H), 1.29 - 1.38 (m, 9H), 2.81 (s, 3 H), 3.04 (m, 3H), 3.75 - 3.94 (m, 2 H), 4.12 - 4.20 (m, 1 H), 4.27 (br s, 3 H), 4.36 - 4.46 (m, 2 H), 4.91 (m, 3 H), 5.00 - 5.22 (m, 1 H), 5.48 - 5.77 (m, 1 H), 6.35 (s, 2 H), 6.98 - 7.26 (m, 3 H), 7.26 - 7.53 (m, 5 H), 7.68 (s, 2 H), 8.33 (s, 1 H), 8.48 - 8.63 (m, 1 H).

$^{31}$P NMR (162 MHz, METHANOL-$d_4$) δ 52.56 (s, 1 P), 56.94 (s, 1 P).

Step 2: To a solution of **S9** (164 mg, 0.125 mmol) in methanol (3.0 mL) was added triethylamine (2.61 mL, 18.8 mmol) in an ice bath. After addition, the mixture was allowed to raise to rt and then concentrated under reduced pressure. The residue was freeze dried. Dichloromethane (0.66 mL) and trifluoroacetic acid (0.66 mL, 8.7 mmol) were mixed first and then added to the triethylamine salt of **S9** (143 mg, 0.097 mmol) at rt. After 7 mins, the solvent was removed under reduced pressure. The residue

was dissolved and concentrated under reduced pressure in toluene (x3) and then acetonitrile (x1). The product was used without further purification. LCMS (AA): m/z = 1177.3 (M+H).

Step 3: To **S10** (B, 118 mg, 0.068 mmol) was added a solution of **S8** (69 mg, 0.096 mmol) in DMF (0.80 mL), followed by N,N-diisopropylethylamine (0.060 mL, 0.34 mmol) at 0 °C. The resulting mixture was warmed to rt over 2h. The reaction mixture was diluted with DMSO (2 mL) and purified on C18 column eluted with 0-50% MeCN-water (with 10 mM NH4OAc). Desired fractions were collected and lyophilized to give 90 mg of the desired product **9** (59% yield). LCMS (AA): m/z = 1779.5 (M+H).

$^{1}$H NMR (400 MHz, METHANOL-D4) δ 0.97 (m, 6 H), 1.34 (m, 2 H), 1.47 (d, J=I.09 Hz, 3 H) 1.52- 1.61 (m, 2 H) 1.63 - 1.75 (m, 1 H) 1.77-1.88 (m, 1 H) 2.11 -2.24 (m, 1 H) 2.44 - 2.59 (m, 2 H) 2.94 (br s, 3 H) 3.35 (s, 3 H) 3.45 (m, 2 H) 3.49 - 3.56 (m, 2 H) 3.58 - 3.66 (m, 28 H) 3.73 (t, J= 6.05 Hz, 2 H) 4.01 (dd, J=I 1.25, 3.42 Hz, 1 H) 4.20 (d, J= 6.85 Hz, 1 H), 4.29 (s, 1 H) 4.33 - 4.59 (m, 5 H) 4.86 (m, 2 H) 4.96 - 5.08 (m, 3 H) 5.15 - 5.30 (m, 1 H) 5.72 (d, J= 52.0 Hz, 1 H) 6.35 - 6.53 (m, 2 H) 6.77 (s, 2 H), 7.09 - 7.24 (m, 2 H) 7.26 - 7.35 (m, 1 H) 7.38 (s, 1 H) 7.43 - 7.59 (m, 4 H), 7.76 (s, 1 H) 7.80 (br d, J=7.46 Hz, 1 H) 8.42 (s, 1 H) 8.62 - 8.69 (s, 1 H).

$^{31}$P NMR (162 MHz, METHANOL-D4) δ 52.26 (s, 1 P) 56.88 (s, 1 P).

**Scheme S4**

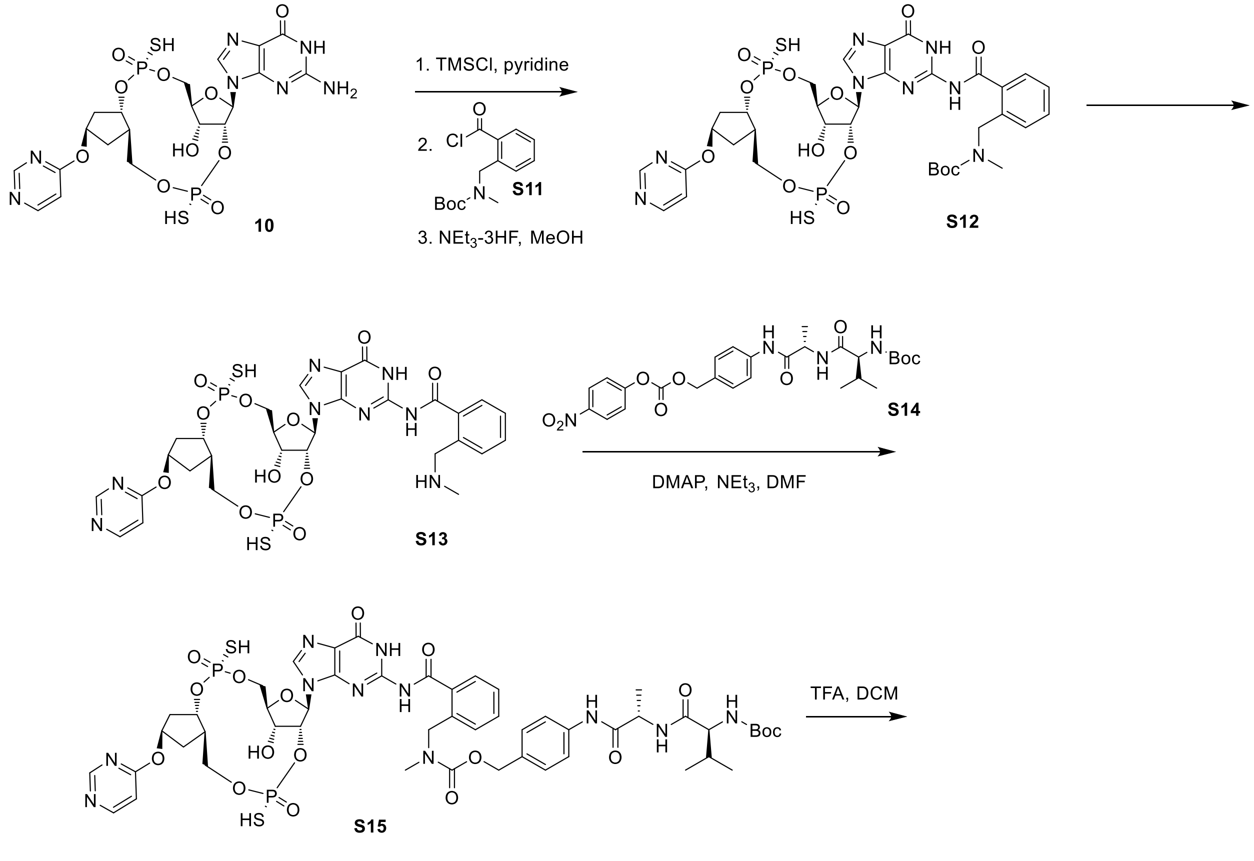

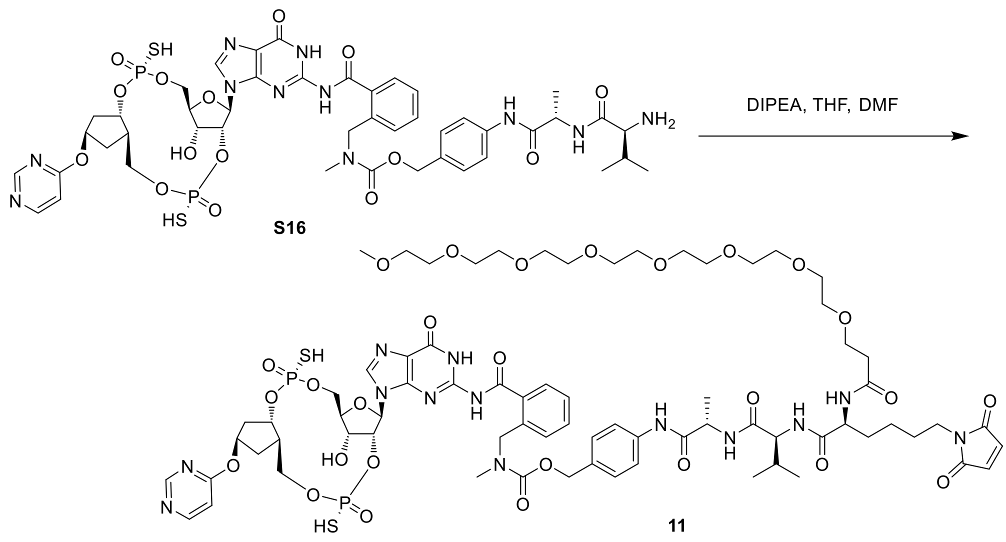


Step 1: **Compound 10** (160 mg, 0.19 mmol) was dissolved in dry pyridine and concentrated to dryness (3 x 2mL) and then placed under vacuum for 15 min. The residue was dissolved in pyridine (3 mL) under an argon atmosphere and chlorotrimethylsilane (0.15 mL, 1.13 mmol) was added. The reaction mixture was allowed to stir at rt for 30 min. **S11** (800 mg, 2.82 mmol) dissolved in pyridine (3 mL) was then added via syringe. The reaction mixture was stirred at rt under an argon atmosphere for 16 h. The reaction mixture was then concentrated to dryness and MeOH (10 mL) and ammonium hydroxide (28-30% solution in water, 10 mL) were added and allowed to stir for 30 min. The reaction mixture was concentrated to dryness and the residue was dissolved in MeOH (15 mL). Triethylamine trihydrofluoride (0.12 mL, 0.75 mmol) was added and the reaction mixture was stirred at rt for 30 min. The reaction mixture was concentrated to dryness and the crude residue was adsorbed onto Celite and purified by reverse phase flash column chromatography (0-50% ACN/ aqueous triethylammonium acetate (10 mM)) to provide **S12** as the N,N-diethylethanamine salt (120 mg, 58%). LCMS (AA): m/z = 897.3 (M+H). 1H NMR (400 MHz, $CD_3OD$) δ 8.70 (s, 1H), 8.49 (s, 1H), 8.41 (d, J = 6.0 Hz, 1H), 7.70 (d, J = 7.5 Hz, 1H), 7.58 – 7.52 (m, 1H), 7.44 – 7.39 (m, 1H), 7.33 (d, J = 7.8 Hz, 1H), 6.85 (d, J = 6.0 Hz, 1H), 6.17 (d, J = 8.3 Hz, 1H), 5.64 – 5.58 (m, 1H), 5.49 – 5.44 (m, 1H), 5.08 – 5.02 (m, 1H), 4.88 – 4.78 (m, 1H), 4.76 – 4.67 (m, 2H), 4.37 – 4.21 (m, 3H), 4.06 – 4.00 (m, 1H), 3.83 – 3.74 (m, 1H), 2.82 (s, 3H), 2.60 – 2.32 (m, 4H), 1.54 – 1.46 (m, 1H), 1.42 (s, 9H). 31P NMR (162 MHz, CD3OD) δ 55.19 (s, 1P), 53.20 (s, 1P).

Step 2: **S12** as the N,N-diethylethanamine salt (50 mg, 0.045 mmol) was added to a round bottom flask and cooled to 0 °C. A solution of trifluoroacetic acid (0.24 mL, 3.2 mmol) and DCM (0.58 mL) was then added via syringe and the reaction mixture was stirred at 0 °C for 30 min. The reaction mixture was then concentrated to dryness and placed under vacuum for 2 h to provide **S13** as the 2,2,2-trifluoroacetate salt (41 mg, 100%). LCMS (AA): m/z = 797.1 (M+H).

Step 3: To a solution of **S14** (58 mg, 0.10 mmol) and 4-dimethylaminopyridine (12 mg, 0.10 mmol) in DMF (0.38 mL) and triethylamine (0.055 mL, 0.40 mmol) was added a solution of **S13** (45 mg, 0.05 mmol) in DMF (1.5 mL) at rt. The reaction mixture was allowed to stir at rt for 15 min. Celite was added and the mixture was concentrated to dryness. The crude residue absorbed onto Celite was purified by reverse phase flash column chromatography (0-40% ACN/aqueous triethylammonium acetate (10 mM)) to provide **S15** as the N,N-diethylethanamine salt (Intermediate 7, 10 mg, 16%). LCMS (AA): m/z = 1216.3 (M+H). 1H NMR (400 MHz, $CD_3OD$) δ 8.70 (s, 1H), 8.43 (brs, 1H), 8.40 (d, J = 5.9 Hz, 1H), 7.70 (d, J = 7.5

Hz, 1H), 7.56 – 7.46 (m, 3H), 7.42 – 7.38 (m, 1H), 7.32 – 7.19 (m, 3H), 6.84 (d, J = 5.5 Hz, 1H), 6.15 (d, J = 7.3 Hz, 1H), 5.62 – 5.56 (m, 1H), 5.54 – 5.48 (m, 1H), 5.08 – 5.02 (m, 1H), 5.04 (s, 2H), 4.88 – 4.83 (m, 1H), 4.81 – 4.76 (m, 2H), 4.53 – 4.47 (m, 1H), 4.37 – 4.22 (m, 3H), 4.08 – 4.03 (m, 1H), 3.92 – 3.89 (m, 1H), 3.82 – 3.74 (m, 1H), 2.88 (s, 3H), 2.58 – 2.30 (m, 4H), 2.10 – 2.03 (m, 1H), 1.54 – 1.47 (m, 1H), 1.46 – 1.44 (m, 3H), 1.44 (s, 9H), 0.98 (d, J = 6.8 Hz, 3H), 0.93 (d, J = 6.8 Hz, 3H). 31P NMR (162 MHz, $CD_3OD$) δ 55.01 (s, 1P), 53.03 (s, 1P).

Step 4: To a flask containing **S15** (56.0 mg, 0.0375 mmol) was added a mixture of DCM (1.13 mL) and TFA (1.11 mL, 14.6 mmol). The mixture was stirred at rt for 5 min. The reaction was concentrated under reduced pressure, then further co-evaporated with dry toluene three more times. The resulting product **S16** was used without further purification. LCMS (AA): m/z = 1116.3 (M+H).

Step 5: To **S16** (115 mg, 0.080 mmol) in a vial was added a solution of **S8** (80 mg, 0.112 mmol) in anhydrous DMF (2.5 mL) followed by N,N-diisopropylethylamine (0.140 mL, 0.80 mmol). The mixture was stirred at room temperature for 30 minutes. The reaction mixture was purified on C18 column eluted with 0-50% MeCN-water (with 10 mM NH4OAc). Desired fractions were collected and lyophilized to give 13 mg of the desired product **11** (9.2% yield).

$^1$H NMR (400 MHz, METHANOL-$d_4$) δ 0.87 (dd, *J*=8.86, 6.91 Hz, 7 H), 1.15 - 1.30 (m, 3 H), 1.37 (d, *J*=7.09 Hz, 3 H), 1.43 - 1.50 (m, 2 H), 1.52 - 1.64 (m, 1 H), 1.66 - 1.80 (m, 1 H), 1.83 (d, *J*=5.14 Hz, 1 H), 2.06 (d, *J*=6.97 Hz, 1 H), 2.40 (br d, *J*=2.93 Hz, 2 H), 2.84 (br s, 3 H), 3.34 (br t, *J*=6.91 Hz, 2 H), 3.40 - 3.47 (m, 3 H), 3.49 - 3.56 (m, 28 H), 3.62 (s, 2 H), 3.86 - 3.98 (m, 1 H), 4.10 (d, *J*=6.85 Hz, 1 H), 4.19 (br d, *J*=1.34 Hz, 1 H), 4.22 - 4.48 (m, 7 H), 4.92 (s, 3 H), 5.03 - 5.18 (m, 1 H), 5.48 - 5.74 (m, 1 H), 6.27 - 6.41 (m, 2 H), 6.67 (s, 2 H), 6.98 - 7.25 (m, 3 H), 7.28 (d, *J*=1.59 Hz, 1 H), 7.31 - 7.48 (m, 4 H), 7.65 (s, 1 H), 7.68 - 7.75 (m, 1 H), 8.31 (s, 1 H), 8.55 (s, 1 H).

$^{31}$P NMR (162 MHz, METHANOL-$d_4$) δ 52.24 (s, 1 P), 56.88 (s, 1 P).

NMR/LCMS LCMS (AA): m/z = 1775.8 (M+H); HRMS (m/z): [M+H]+ calcd for C75H104N14O28P2S2 1775.6134; found, 1775.6138.

**Scheme S5**

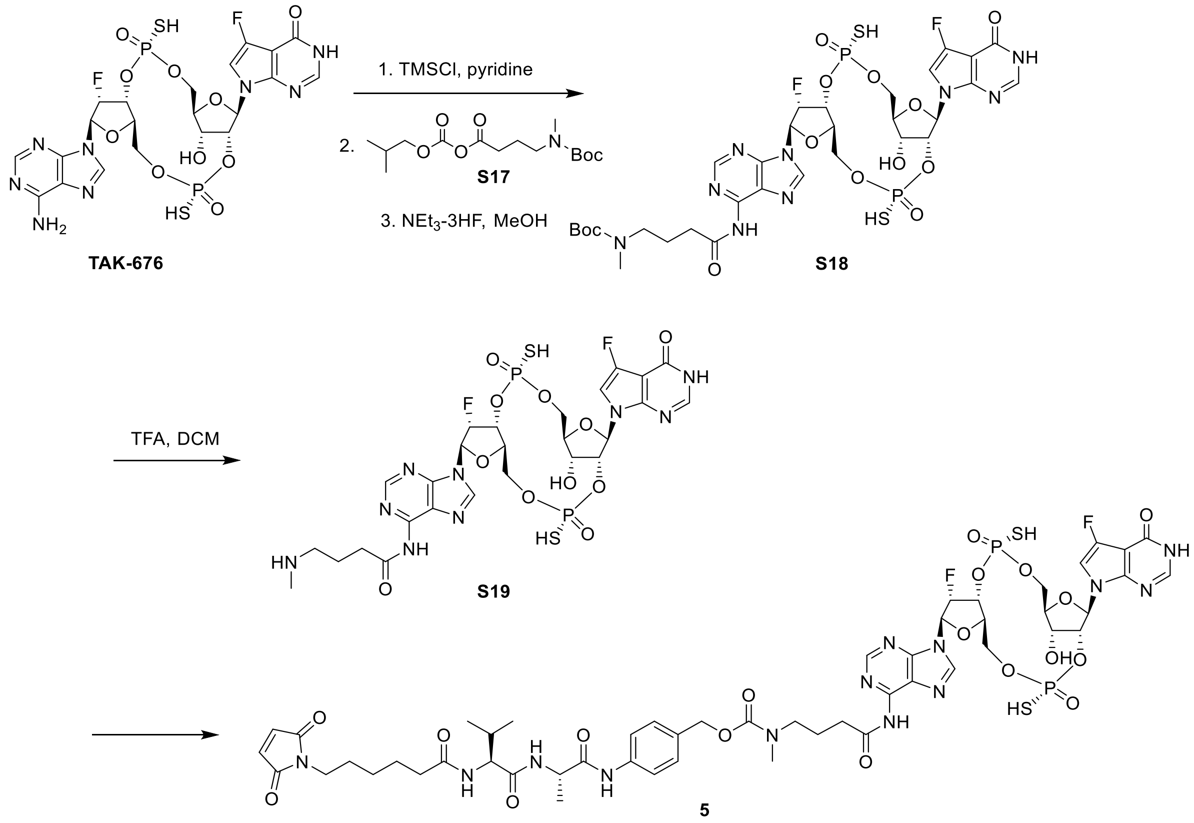


Step 1:

To a 50-mL RBF was added dazostinag (243 mg, 0.266 mmol), pyridine (3.0 mL), and TMSCl (0.276 mL, 2.13 mmol). The mixture was stirred at rt for 2h. **S17** (503 mg, 1.59 mmol) in pyridine (3.2 mL) was added to the mixture in an ice bath. After addition, the mixture was slowly warmed to rt and stirred for 12h. The reaction mixture was cooled to 0 °C and then ammonium hydroxide (20.0 mL, 598 mmol) was slowly added. The mixture was warmed to rt and stirred for 2h. Purification by reverse phase flash column chromatography (0-50% ACN/aqueous ammonium bicarbonate (5 mM)) gave **S18** as the ammonium salt (148 mg, 59%). LCMS (AA): m/z = 910.5 (M+H). 1H NMR (400 MHz, D2O) δ 8.70 (s, 1H), 8.34 (s, 1H), 8.01 (s, 1H), 7.28 (s, 1H), 6.56 (d, J = 16.1 Hz, 1H), 6.43 (d, J = 8.4 Hz, 1H), 5.67 (dd, J = 51.0, 3.9 Hz, 1H), 5.18 – 4.99 (m, 2H), 4.82 – 4.77 (m, 1H), 4.63 – 4.58 (m, 1H), 4.49 – 4.38 (m, 3H), 4.31 – 4.25 (m, 1H), 4.12 – 4.06 (m, 1H), 3.43 – 3.36 (m, 2H), 2.88 (s, 3H), 2.73 – 2.68 (m, 2H), 2.04 – 1.97 (m, 2H), 1.34 (s, 9H). 31P NMR (162 MHz, D2O) δ 55.47 (s, 1P), 52.07 (s, 1P). 19F NMR (376 MHz, CD3OD) δ -165.32 to -165.67 (m, 1F), -203.24 to -203.65 (m, 1F).

Step 2:

A solution of **S18** as the ammonium salt (42 mg, 0.045 mmol) in TFA (0.24 mL) and DCM (0.58 mL) was stirred at rt for 30 min. Concentration provided the desired product **S19** (41 mg, 100%). The intermediate was used immediately in the next reaction. LCMS (AA): m/z = 810.2 (M+H).

Step 3: C-21

To a solution of **S19** (0.1397 mmol, 173 mg) N-succinimidyl 6-maleimidohexanoate (98.0 mg, 0.318 mmol) in DMF (3.3 mL) was added N,N-diisopropylethylamine (0.15 mL, 0.86 mmol). The reaction mixture was stirred at rt for 2 h. The reaction mixture was diluted with DMSO (2 mL) and purified on C18 column (275g) eluted with 0-50% MeCN-water (with 10 mM $NH_4OAc$). Desired fractions were collected and lyophilized to give 94 mg product **5** (49% yield).

$^{1}$H NMR (400 MHz, DMSO-$d_6$) δ 0.84 (dd, *J*=16.14, 6.72 Hz, 6 H), 1.20 (br d, *J*=7.70 Hz, 3 H), 1.31 (d, *J*=7.09 Hz, 3 H), 1.48 (br s, 4 H), 1.79 - 1.91 (m, 2 H), 1.97 - 2.04 (m, 1 H), 2.06 - 2.26 (m, 2 H), 2.62 (s, 2 H), 2.87 (br s, 3 H), 3.25 - 3.38 (m, 6 H), 3.68 - 3.78 (m, 1 H), 3.95 - 4.07 (m, 1 H), 4.11 - 4.28 (m, 4 H), 4.32 - 4.49 (m, 2 H), 4.53 - 4.66 (m, 1 H), 4.98 (s, 3 H), 5.10 - 5.31 (m, 1 H), 5.67 - 5.92 (m, 1 H), 6.25 - 6.32 (m, 1 H), 6.34 - 6.44 (m, 1 H), 6.94 - 7.06 (m, 4 H), 7.10 (br s, 2 H), 7.23 (br s, 4 H), 7.57 (br s, 3 H), 7.74 - 7.84 (m, 1 H), 7.91 (d, *J*=3.79 Hz, 1 H), 8.07 - 8.21 (m, 1 H), 8.53 (s, 1 H), 8.60 - 8.71 (m, 1 H), 9.80 - 10.06 (m, 1 H), 10.62 - 10.74 (m, 1 H), 12.08 (br d, *J*=3.06 Hz, 1 H).

$^{31}$P NMR (162 MHz, DMSO-$d_6$) δ 48.15 (s, 1 P), 54.08 (s, 1 P).

LCMS (AA): m/z = 1322.3 (M+H); HRMS (m/z): [M+H]+ calcd for C52H63F2N13O18P2S2 1322.3371; found, 1322.3394.

**Scheme S6**

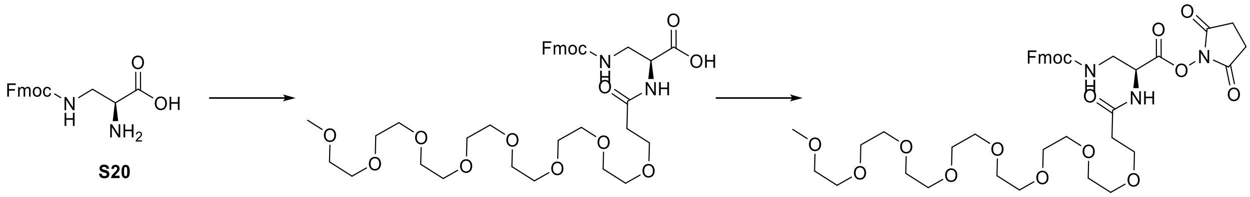

S20 S21 S22

Step 1: To a flask containing **S20** (1.35 g, 3.07 mmol) was added DCM (30 mL) and N,N-diisopropylethylamine (2.2 mL, 12.3 mmol, 100). Methyl-PEG8-NHS ester (1.7 g, 3.37 mmol) was added to the solution and the resulting mixture was stirred at rt for 2h. Purification on C18 column eluted with 0-50% MeCN-water (with 10 mM formic acid) provided 1.9 g product **S21** (2.6 mmol, 86% yield). LCMS (AA): m/z = 721.5 (M+H). $^{1}$H NMR (400 MHz, DMSO-$d_6$) δ 2.31 (s, 5 H), 2.36 - 2.42 (m, 1 H), 3.24 (s, 2 H), 3.40 - 3.46 (m, 4 H), 3.47 - 3.55 (m, 22 H), 3.59 (br t, *J*=6.54 Hz, 2 H), 4.28 (br s, 2 H), 7.13 - 7.20 (m, 4 H), 7.25 (d, *J*=7.34 Hz, 3 H), 7.34 (s, 1 H), 7.43 (s, 1 H), 7.69 (br d, *J*=7.21 Hz, 1 H), 7.90 (d, *J*=7.46 Hz, 1 H).

Step 2: To a flask containing a film of **S21** (364 mg, 0.505 mmol) was added N-hydroxysuccinimide (58 mg, 0.505 mmol), N,N'-dicyclohexylcarbodiimide (99 mg, 0.500 mmol) and DCM (3.2mL). Precipitation started to form immediately. The suspension was stirred at room temperature for 90 minutes. The reaction was cooled in an ice bath for 30 minutes and filtered. The filtrate was concentrated and purified on a silica gel column (eluted with 0-10% acetone in DCM) to provide **S22** (126 mg, 35% yield). The product was used immediately in the next step. $^{1}$H NMR (400 MHz, DMSO-$d_6$) δ 2.41 (br t, *J*=6.54 Hz, 2 H), 2.60 (s, 2 H), 2.82 (s, 5 H), 3.24 (s, 4 H), 3.45 - 3.53 (m, 22 H), 3.60 (br t, *J*=6.30 Hz, 3 H), 3.91 (s, 2 H),

4.17 - 4.45 (m, 3 H), 4.75 - 4.96 (m, 1 H), 7.29 - 7.37 (m, 2 H), 7.39 - 7.51 (m, 3 H), 7.67 (br d, *J*=4.77 Hz, 2 H), 7.89 (d, *J*=7.58 Hz, 2 H), 8.55 (d, *J*=8.07 Hz, 1 H), 10.50 - 10.65 (m, 1 H).

**Scheme S7**

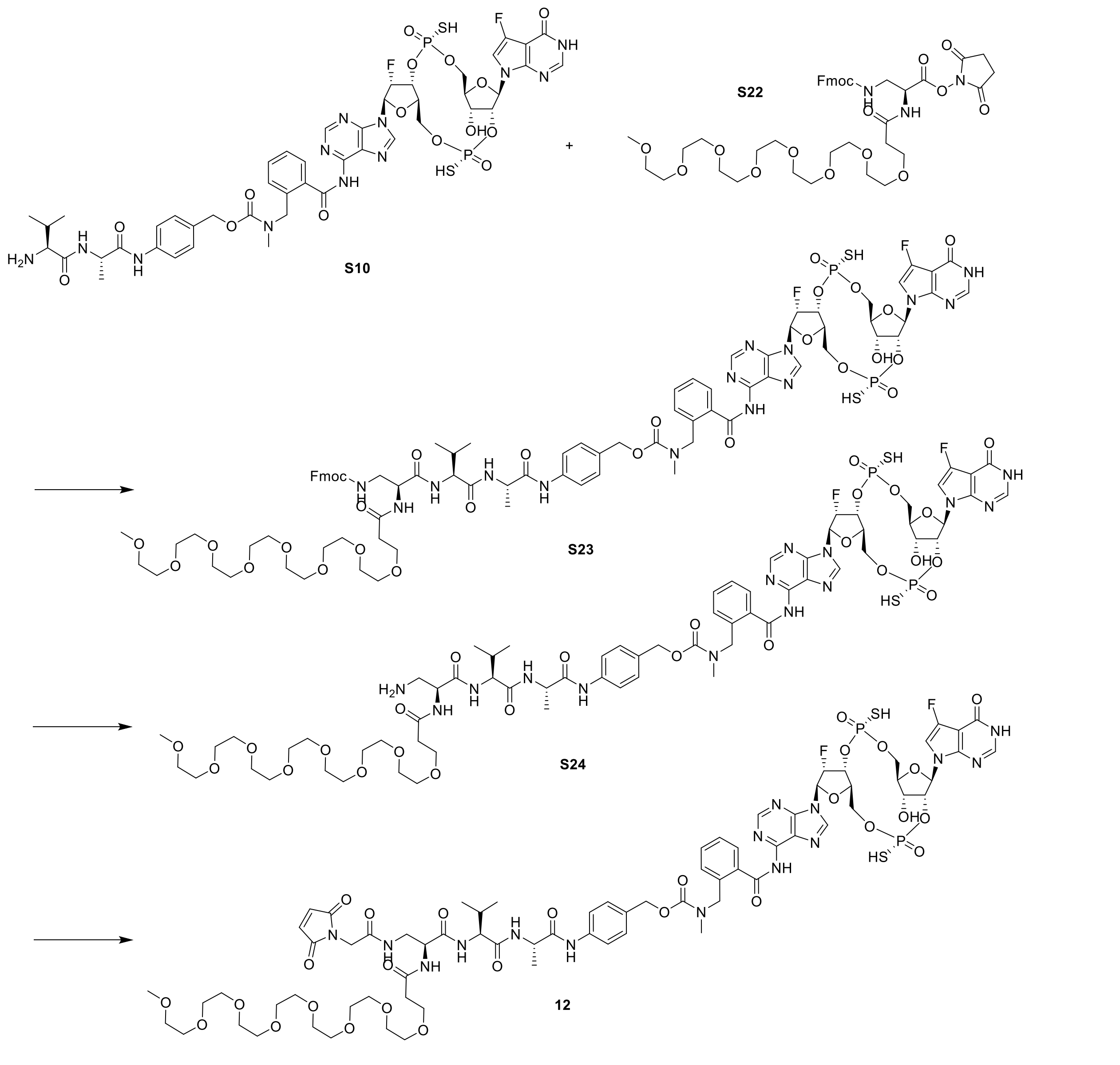


Step 1: To a solution of **S22** (126 mg, 0.155 mmol) and **S10** (233 mg, 0.135 mmol) in DMF (2.5 mL) was added DIEA (0.35 mL, 2.03 mmol). The reaction was stirred at rt for 30min. The reaction mixture was purified on C18 column eluted with 0-50% MeCN-water (with 10 mM $NH_4OAc$) to provide the desired product **S23** (93 mg, 0.049 mmol, 36% yield). LCMS (AA): m/z = 1879.0 (M+H). $^1$H NMR (400 MHz, DMSO-$d_6$) δ 0.84 (br dd, *J*=18.65, 6.54 Hz, 6 H), 1.30 (br d, *J*=6.97 Hz, 3 H), 1.89 (s, 2 H), 2.03 (br d, *J*=6.97 Hz, 1 H), 2.37 (br t, *J*=6.36 Hz, 4 H), 2.83 (s, 3 H), 3.21 (s, 3 H), 3.37 - 3.42 (m, 3 H), 3.41 - 3.51 (m, 24 H), 3.57 (t, *J*=6.42 Hz, 1 H), 3.67 - 3.75 (m, 1 H), 3.92 (br d, *J*=8.19 Hz, 1 H), 4.03 - 4.14 (m, 2 H), 4.15 - 4.27 (m, 4 H), 4.30 - 4.47 (m, 3 H), 4.58 (d, *J*=3.79 Hz, 1 H), 4.69 (br s, 2 H), 4.90 - 5.08 (m, 3 H), 5.11 - 5.25 (m,

1 H), 5.64 - 5.95 (m, 1 H), 6.20 - 6.32 (m, 1 H), 6.36 (br d, *J*=13.94 Hz, 1 H), 7.03 - 7.33 (m, 7 H), 7.32 - 7.40 (m, 3 H), 7.43 - 7.73 (m, 9 H), 7.77 - 7.94 (m, 4 H), 7.95 - 8.09 (m, 2 H), 8.21 (br d, *J*=6.85 Hz, 3 H), 8.57 (s, 3 H), 9.87 - 10.03 (m, 1 H).

Step 2 : To a flask containing **S23** (46.0 mg, 0.0245 mmol) was added 20% piperidine in DMF (4.89 mmol) and stir for 20 minutes. The reaction mixture was purified on C18 column eluted with 0-50% MeCN-water (with 10 mM $NH_4OAc$) to provide the desired product **S24** (23.0mg, 0.0136 mmol, 55% yield). LCMS (AA): m/z = 1657.6, 1658.8 (M+H). $^1$H NMR (400 MHz, DMSO-$d_6$) δ 0.74 - 0.97 (m, 6 H), 1.31 (br d, *J*=6.36 Hz, 3 H), 1.92 (s, 2 H), 2.10 (br d, *J*=6.60 Hz, 2 H), 2.43 (br t, *J*=6.48 Hz, 4 H), 2.87 (s, 2 H), 2.92 - 3.02 (m, 1 H), 3.04 - 3.11 (m, 1 H), 3.24 (s, 5 H), 3.44 (br d, *J*=5.01 Hz, 3 H), 3.45 - 3.55 (m, 24 H), 3.61 (t, *J*=6.36 Hz, 1 H), 3.75 (br d, *J*=12.10 Hz, 1 H), 3.97 (br d, *J*=8.44 Hz, 1 H), 4.11 - 4.24 (m, 3 H), 4.37 (br s, 2 H), 4.53 - 4.64 (m, 2 H), 4.67 - 4.75 (m, 2 H), 4.83 (br s, 1 H), 4.99 (br s, 3 H), 5.16 - 5.34 (m, 1 H), 5.71 - 6.03 (m, 1 H), 6.30 (br d, *J*=7.95 Hz, 1 H), 6.35 - 6.44 (m, 1 H), 7.02 - 7.30 (m, 4 H), 7.39 (br t, *J*=7.46 Hz, 2 H), 7.46 - 7.70 (m, 6 H), 7.76 - 7.97 (m, 3 H), 8.18 - 8.28 (m, 1 H), 8.30 - 8.43 (m, 1 H), 8.59 (s, 2 H), 9.92 - 10.09 (m, 1 H).

Step 3 : To a solution of the **S24** (23.0 mg, 0.0136 mmol) in DMF (1.0 mL) was added maleimidoacetic acid N-hydroxysuccinimide ester (0.0163 mmol) and N,N-diisopropylethylamine (0.204 mmol, 0.20 mmol). The reaction was stirred at rt for 20 minutes. The reaction mixture was purified on C18 column eluted with 0-50% MeCN-water (with 10 mM $NH_4OAc$). Desired fractions were collected and lyophilized to give 4.2 mg of the desired product **12** (17% yield). LCMS (FA): m/z = 1794.5 (M+H).

$^1$H NMR (400 MHz, DMSO-$d_6$) δ 0.61 - 0.97 (m, 6 H), 1.25 (br d, *J*=6.97 Hz, 3 H), 1.88 - 2.06 (m, 1 H), 2.28 - 2.35 (m, 2 H), 2.78 (br s, 3 H), 3.16 (s, 3 H), 3.36 (br d, *J*=5.14 Hz, 3 H), 3.39 - 3.46 (m, 25 H), 3.52 (br t, *J*=6.54 Hz, 2 H), 3.65 (br d, *J*=13.82 Hz, 1 H), 3.81 - 3.91 (m, 1 H), 3.90 - 4.01 (m, 2 H), 4.01 - 4.09 (m, 2 H), 4.10 - 4.19 (m, 2 H), 4.26 - 4.42 (m, 3 H), 4.52 (br s, 1 H), 4.64 (br s, 2 H), 4.73 (br s, 1 H), 4.87 - 5.05 (m, 3 H), 5.14 (br d, *J*=3.18 Hz, 1 H), 6.20 (br d, *J*=7.83 Hz, 1 H), 6.31 (br dd, *J*=14.00, 3.36 Hz, 1 H), 6.43 (s, 1 H), 6.92 - 7.02 (m, 3 H), 7.31 (br t, *J*=7.40 Hz, 1 H), 7.39 - 7.61 (m, 5 H), 7.71 (br d, *J*=8.31 Hz, 1 H), 7.81 (s, 1 H), 7.97 (br d, *J*=8.07 Hz, 1 H), 8.02 - 8.12 (m, 1 H), 8.20 (br d, *J*=6.85 Hz, 1 H), 8.51 (s, 2 H), 9.94 (s, 1 H), 11.07 - 11.41 (m, 1 H), 11.81 - 12.16 (m, 1 H).

$^{31}$P NMR (162 MHz, DMSO-$d_6$) δ 46.94 (s, 1 P), 53.63 (s, 1 P).

**Bioconjugation**

Analytical SEC conditions:

SEC spectra were recorded on a Hewlett-Packard HP1100 or an Agilent 1100 Series LC system with Diode Array Detector using a SEC column (typically Tosoh Biosep TSK Gel, G3000SWxl; P/N 8541; 250A; 5um; 7.8mm x 300mm) at 280 nm. Mobile phase was 100 mM sodium phosphate, 300 mM sodium chloride, pH 6.8, 10% acetonitrile (v/v) or 1xPBS. A typical run is isocratic at a flow rate of 1 mL/min for 20 min.

Analytical HIC conditions:

HIC spectra were recorded on a Hewlett-Packard HP1100 or Agilent 1100 Series LC system with Diode Array Detector using a HIC column (typically Tosoh Butyl-NPR, 4.6 x 35 mm, 2.5 um, P/N: 14947) at 280 nm. Mobile phase A was 25 mM sodium phosphate, 1.5 M ammonium sulfate, pH 7, and Mobile phase B

was 75% 25 mM sodium phosphate, pH 7, 25% isopropanol. For a typical 20 min run, a 12 min linear gradient from 95%/5% A/B to 100%B would be used between initial and final intervals of isocratic flow.

LC-QTOF conditions:

LCMS spectra were recorded on an Agilent 1260 Bioinert Series LC system connected to an Agilent 6545 QTOF mass spectrometer using a reverse phase column heated to 80 °C (typically Agilent, PLRP-S, 5 µm, 1000 Å, 2.1 mm x 50 mm). Various gradients and run times were selected in order to best characterize the compounds. Mobile phases were based on ACN/water gradients and contained 0.1% formic acid. One example of a solvent gradient that was used was 95% mobile phase A (mobile phase A = 99% water + 1% ACN + 0.1% formic acid) to 100% mobile phase B (mobile phase B = 95% ACN + 5% water + 0.1% formic acid) with conditions shown in Table S1.

Table S1

| Time (min) | Flow (mL/min) | %A | %B |
|---|---|---|---|
| 0 | 0.35 | 82 | 18 |
| 1 | 0.35 | 82 | 18 |
| 2 | 0.35 | 70 | 30 |
| 19 | 0.5 | 50 | 50 |
| 19.5 | 0.5 | 10 | 90 |
| 21 | 0.5 | 10 | 90 |
| 21.1 | 0.5 | 82 | 18 |
| 22 | 0.5 | 82 | 18 |

Samples were either intact or reduced (20 uL of 1~5 mg/mL ADC solution treated with 4 uL of 0.5M DTT solution at 37 °C for 30 min). Raw data was deconvoluted within appropriate mass range using Agilent BioConfirm software to obtain protein molecular weight(s), and the Agilent DAR Calculator was used to calculate DAR.

LC/MS/MS conditions:

LC/MS/MS analysis was performed using Shimadzu UFLC LC-20AD XR binary pump and SIL-30AC MP autosampler system and AB SCIEX Triple Quad 4500 ESI Mass spectrometry.

Typically, 5 uL sample aliquots were injected into the LC/MS/MS after passing through a Waters X select C18 CSH 3.5u 2.1 mm ID x 30 mm column. Mobile phase A contained 0.1% formic acid in water, and mobile phase B contained 0.1% formic acid in 5% water with 95% acetonitrile. Total run time was 3 min at 1.5 mL/min with a linear gradient from 100% A to 100% B over 1.5 min flow rate. Initially, the instrument was running at 100% aqueous mobile phase solvent for 0.5 min, and then it was increased to 100% organic solvent in next 1.5 min.

Preparative SEC:

Preparative SEC purification was conducted on a Gilson Preparative HPLC system with UV Detector using a SEC column (typically GE Superdex 200 Increase 10/300 GL). Mobile phase was 1xPBS (pH 7.4). A typical run was isocratic at a flow rate of 1 mL/min for 30 min. Fraction collection was triggered based on UV threshold (at 214 and 280 nm).

ADC concentration:

ADC concentration was calculated from the UV absorbance at 280 nm measured by NanoDrop (2000c; Fisher Scientific) coefficient after subtraction of the UV absorbance from the corresponding linker-payload constructs.

TAK-500 Preparation

TCEP (1 mM solution in water, 2.5 equiv.) was added to a solution of an anti-CCR2 antibody (TAK-202, 10 mg/mL, generated as described in US 7,473,421 B2) in 50 mM histidine, 125 mM arginine, pH 6.1 buffer. The reaction mixture was purged with argon and incubated at room temperature (RT) for 3 hours with gentle shaking. The linker-payload construct (5 mM solution in DMA, 7 equiv.) was then added slowly into the above mixture. The reaction was purged with argon and incubated at RT for another 1 hour with gentle shaking. The reaction mixture was purified on a Gilson Preparative high performance liquid chromatography (HPLC) system with ultraviolet (UV) detector using a size exclusion chromatography (SEC) column (GE Superdex 200 Increase 10/300 GL, 50 mM histidine, 125 mM arginine, pH 6.1 buffer, 1mL/min). The ADC concentration, percentage aggregation, and drug-antibody ratio (DAR) were determined by UV absorbance measured by NanoDrop 2000c, analytical SEC, and analytical hydrophobic interaction chromatography (HIC)/liquid chromatography-tandem mass spectrometry (LC-QTOF) respectively. The average DAR was 3.5~4.5.

mTAK-500 Preparation

To a solution of anti-mCCR2 antibody (RtMuMC-21-S98, 3.4 mg/mL, as described previously (24) in 50 mM histidine, 125 mM arginine, pH 6.1 buffer, was added 0.5M tris, 25mM EDTA, pH 8 buffer to adjust pH to 7. TCEP (10 mM solution in water, 20 equiv.) was then added. The reaction mixture was purged with argon and incubated at 37°C for 1.5 hours with gentle shaking. The resulting mixture was purified on a Gilson Preparative HPLC system with UV detector using a SEC column (GE Superdex 200 Increase 10/300 GL, 50 mM histidine, 125 mM arginine, pH 6.1 buffer, 1mL/min) and concentrated to ~5 mg/mL. Dehydroascorbic acid (2mM in DMSO, 2.2 equiv.) at 4°C was added to the purified antibody, and the resulting mixture was kept at 4°C overnight. The reaction was warmed to RT, and then the linker-payload construct (5 mM solution in DMA, 5 equiv.) was added slowly. The reaction was purged with argon and incubated at RT for 2 hours with gentle shaking. The resulting mixture was purified on a Gilson Preparative HPLC system with UV detector using a SEC column (GE Superdex 200 Increase 10/300 GL, 50 mM histidine, 125 mM arginine, pH 6.1 buffer, 1mL/min). The ADC concentration, percentage aggregation, and DAR were determined by UV absorbance measured by NanoDrop 2000c, analytical SEC, and analytical HIC/LC-QTOF respectively. The average DAR was 2.5~4.0.

**Figure S1**. HIC chromatograms of mCCR2-TAK676 ADC under different reduction conditions after purification. (a) 3 equiv TCEP, 3h, 37 °C. (b) 20 equiv TCEP, 3h, 37 °C. (c) 20 equiv TCEP, 3h, 37 °C, removal of the excess TCEP, then 4.4 equiv DHAA, 4 °C, overnight.

(a)

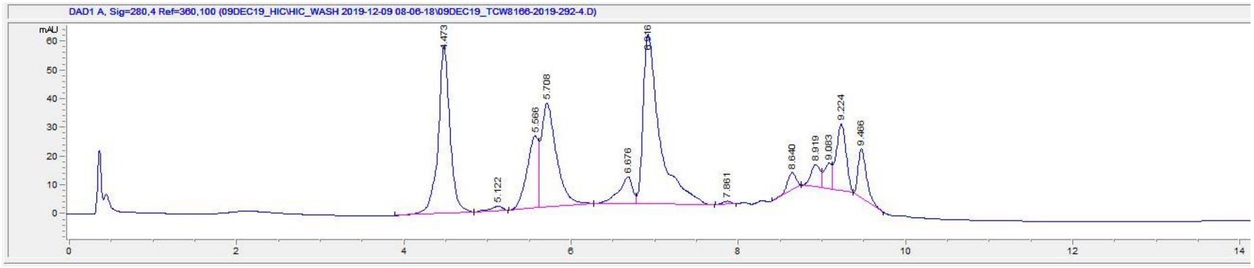


(b)

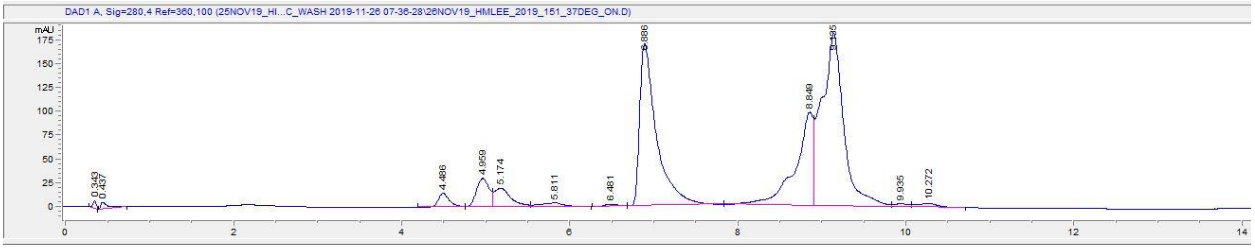


(c)

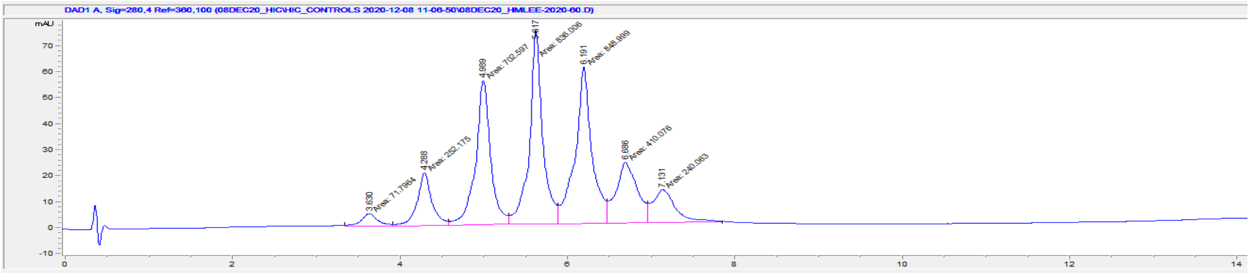


## Safety

No unexpected or unusually high safety hazards were encountered.

## Chemical stability assessment

Powder was weighed out and made up to 4 mg/mL concentration in DMSO. 1:10 dilution of 4 mg/mL DMSO stock solution into 4 HPLC vial containing DMSO, pH 5, and pH 7 phostphate buffers. Then, inject the solution into LC/MS system. DAD peak area and MS were used to monitor any degradation. Samples were incubated at 37 °C and monitored for 7 days.

**Table S2**. Chemical stability of **1** over 7 days

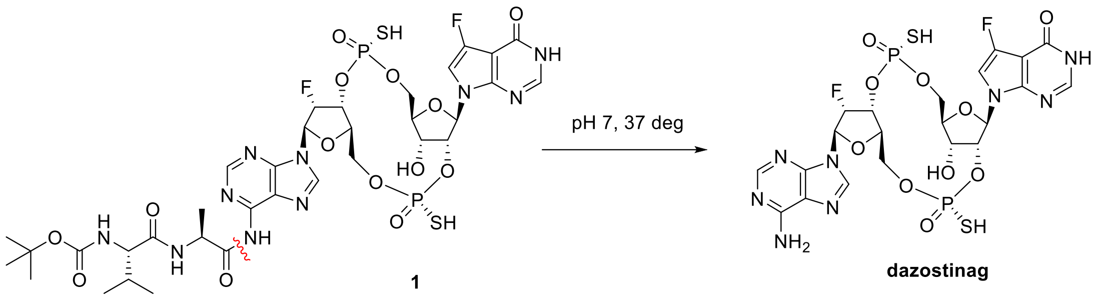


| | Percent purity of **1** (%) | | |
|---|---|---|---|
| Time (days) | DMSO | pH 5 | pH 7 |
| 0 | 100 | 100 | 100 |
| 1 | 100 | 97 | 96 |
| 2 | 100 | 94 | 93 |
| 5 | 100 | 85 | 83 |
| 6 | 100 | 84 | 81 |
| 7 | 100 | 80 | 77 |

**Figure S2.** LC analysis of **1** at pH 7 on day 0 and day 7. The peak at 2.30 min corresponds to **1**, while the peak at 0.76 min corresponds to dazostinag. No other major impurities were observed.

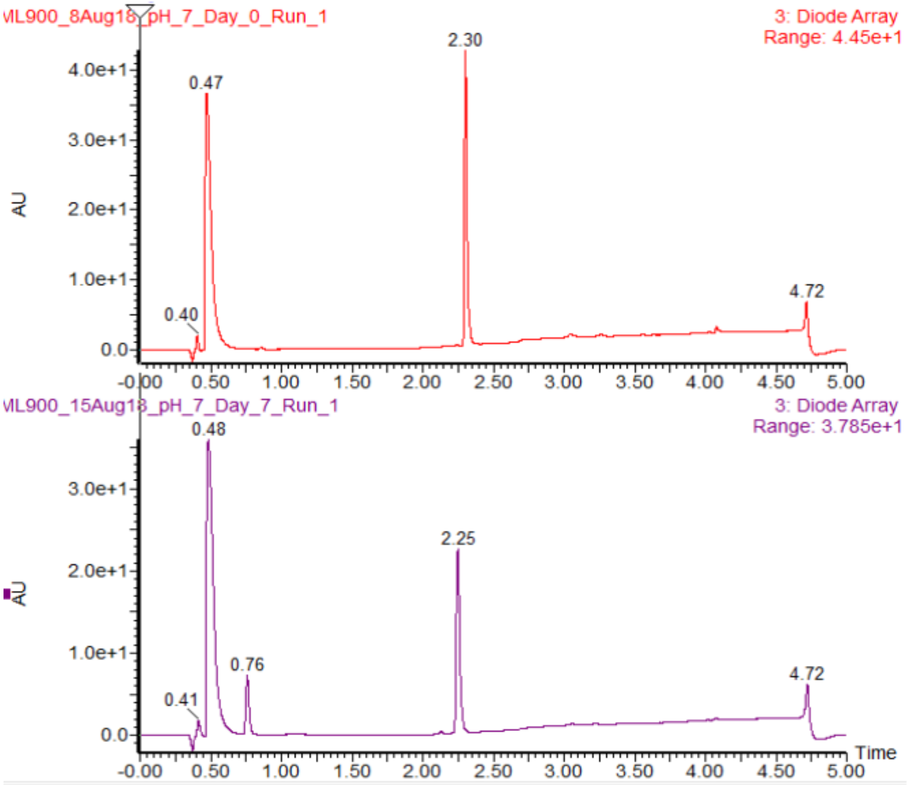

**Table S3.** Chemical stability of **S25**. Multiple unidentified degradants were observed. The observed M+H of the major degradant was 241.

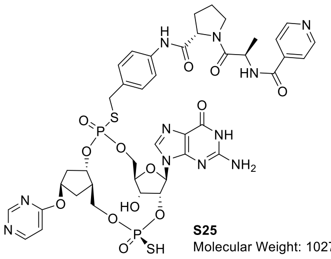

**S25**
Molecular Weight: 1027.96

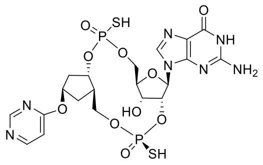

**10**
Molecular Weight: 649.53

| | Percent purity of **S25** (%) | | |
|---|---|---|---|
| Time (days) | DMSO | pH 5 | pH 7 |
| 0 | 97 | 98 | 98 |
| 1 | 95 | 74 | 79 |
| 2 | 95 | 57 | 73 |
| 3 | 95 | 45 | 75 |
| 6 | 94 | 19 | 42 |
| 7 | 93 | 14 | 28 |

**Figure S3.** LC analysis of **S25** at pH 5 on day 0 and day 7. The peak at 2.21 min corresponds to **S25**. Multiple degradants were observed under the peak at 1.63-1.65 min in 7 days.

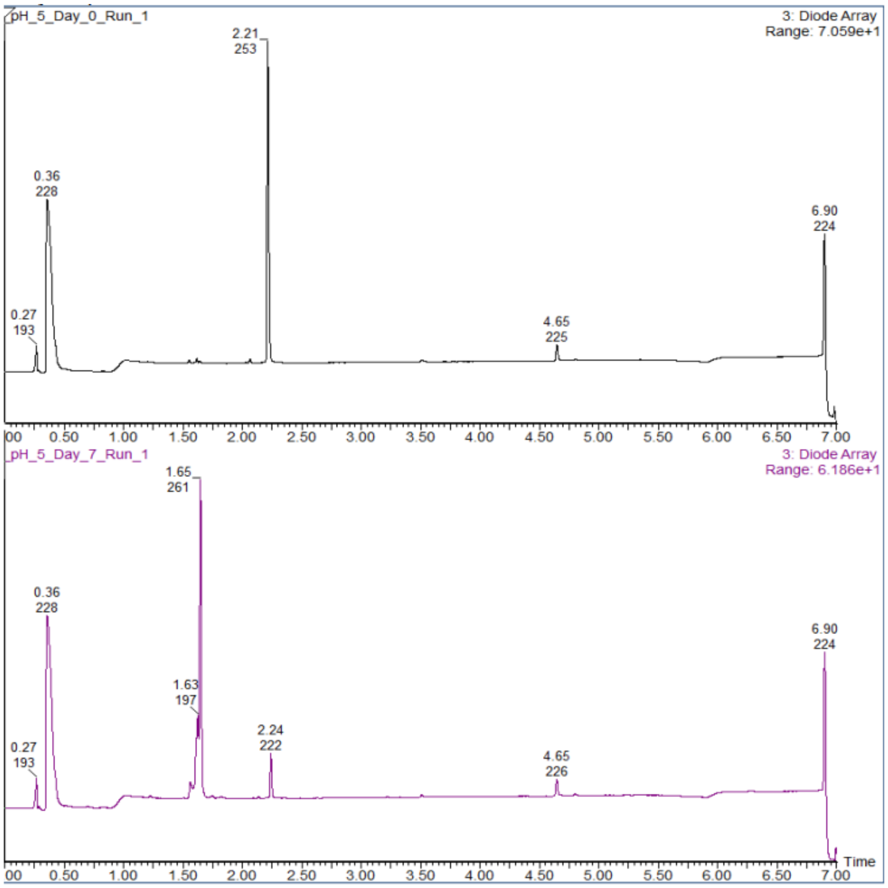

**Tritosomal stability assay**

For payload stability, 121 uM DMSO working solution was made from 10 mM stock solution of payload. Then, the 121uM DMSO working solution was diluted to 1 uM in 557.75 uL solution by preparing a mixture of rat liver tritosomes (purchased from XenoTech) and tritosome buffer solution in which it contained 22.4% rat liver tritosomes. For ADC molecules, mixed 4.65 uL of ADC solution with 557.75 uL of mixture of rat liver tritosomes in tritosome buffer.

Then, the solutions were placed in an incubator and incubated for 24 hours under 37°C. 40 uL of samples were taken out at 10 and 30 minutes, 1, 3, 5, and 24 hours or at only 1, 3, 5 and 24 hours, followed by addition of 160 uL of 0.1% formic acid in methanol solution in a 96-wells plate. Then, the plate was stored in -80 °C freezer. After collecting the last time point, another 200 uL of 0.1% formic acid in methanol solution spiked with 150 nM carbutamide which is an internal standard solution was added into the samples. The samples were mixed well and centrifuged at 4000g for 10 minute, and the 96-wells plate was submitted for LC/MS/MS injection and analysis.

Tritosome buffer solution: Prepared a solution in purified water using 47.9 mg/ml of potassium phosphate monobasic (Sigma-Aldrich Product Number P5379); 6.8 mg/ml of sodium phosphate dibasic (Sigma-Aldrich Product Number S0876), 1.7 mg/ml of ethylenediaminetetraacetic acid (Sigma-Aldrich Product Number ED4SS). pH was adjusted to 6.0 using 1N HCl or 1N KOH.

To monitor the stability of payload, a peak area or concentration verse time curve was plotted. A horizontal curve would be plotted if the payload was stable in rat liver tritosomes. To monitor and calculate the release of payload from ADC, a peak area or concentration verse time curve was plotted. The data would be analyzed by using Excel-Fit program to calculate t1/2 of formation of the payload from the molecules.

**Table S4**. Payload release from **2**, **ADC 2** monitored over time in tritosome

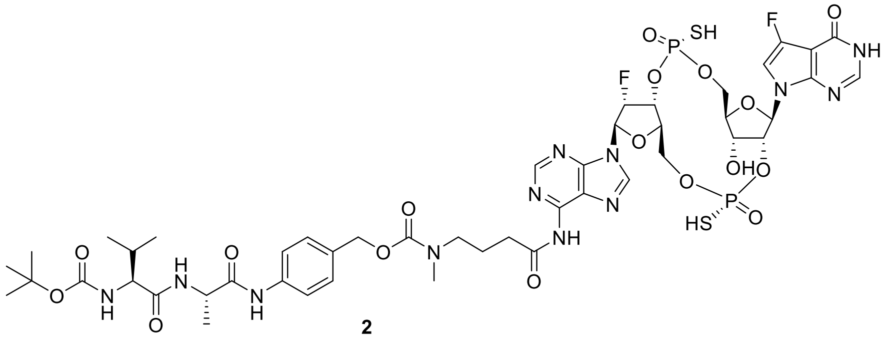

**2**

| Time [hrs] | Payload release in tritosome (nM) |
|---|---|
| 0.03 | 54 |
| 1 | 734 |
| 3 | 660 |
| 5 | 647 |
| 24 | 737 |

theoretical concentration of the payload : 820nM

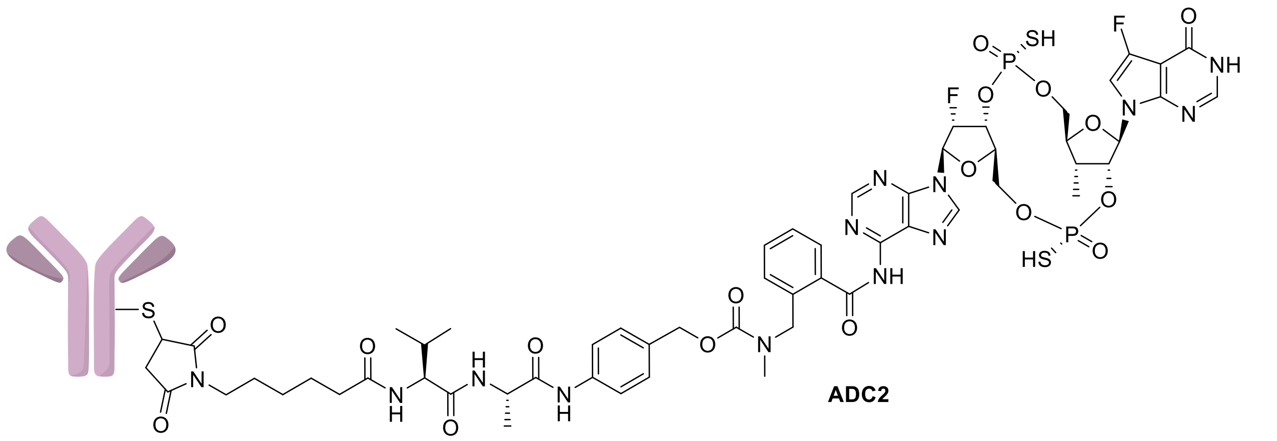


| Time [hrs] | Payload release in tritosome (nM) |
|---|---|
| 0 | 0 |
| 1 | 62 |
| 5 | 189 |
| 24 | 245 |

theoretical concentration of the conjugated payload : 290nM

**Plasma Stability Assay**

Test compounds were spiked into 1 mL of plasma at a concentration of 10 µg/mL and then 5 equal volume aliquots were dispensed into 2 mL Eppendorf microfuge tubes (labeled 0, 24, 48, 72, and 96 hours). The time 0 sample tubes were immediately stored at -80 °C and the remaining tubes were incubated at 37 °C with moderate shaking. Aliquots were removed from the incubator at their corresponding time point and stored at -80 °C. After all samples have been collected, they were thawed at rt and placed on wet ice. 50 µL of each sample was dispensed in triplicate into 96-well microtiter plate. Samples were quenched with 200 µL of ice cold methanol containing 50 nM of internal standard. Samples were vortexed for 2 min then centrifuged at 3000 rpm for 10 min. 185 µL of supernatant was transferred to a clean injection plate then dried down under N2 gas at 40 °C. Dried sample extracts were reconstituted with 100 µL of LCMS grade water then vortexed for 1 min in preparation for LC-MS/MS analysis.

Each sample was separated by reverse phase HPLC using a Synergi 2.5µ Polar-RP 100A C18 column (2.0 mm X 30 mm), (Phenomenex®) at 40 °C using a gradient consisting of 0.1% formic acid in water (Solvent A) and 0.1% formic acid in acetonitrile (Solvent B). Analytes were detected by positive ion spray in multiple-reaction monitoring (MRM) mode using a SCIEX API 4500 QTRAP instrument.

**THP1 Dual Lucia Reporter Gene Assay**

THP1-Dual™ KI-hSTING-R232 cells (InvivoGen #thpd-r232) were derived from the human THP-1 monocyte cell line by stable biallelic knockout of the endogenous human HAQ STING gene and knockin of the R232 variant of human STING. These cells also stably express inducible secreted Lucia luciferase reporter gene under the control of an ISG54 (interferon-stimulated gene) minimal promoter in

conjunction with five IFN-stimulated response elements (ISRE). The expression of reporter gene allows the study of the IFN regulatory factor (IRF) pathway by assessing the activity of Lucia luciferase. In addition to human STING and luciferase, these cells were engineered to stably express human CCR2 to allow the study of target-mediated activation of IRF pathway. The THP-1 cells express endogenous human CCR2 at a much lower density compared to that of the engineered cells to over express human CCR2. Therefore the empty vector cells could still be used as the negative control.

On the day of experiment, the cells were plated to a white, 384-well plate (Corning 356661) at 15,000 cells/25 µL per well density in growth media (RPMI 1640, 2 mM L-glutamine, 25 mM HEPES, 10% heat-inactivated fetal bovine serum, 100 µg/mL Normocin™, 100 U/mL-100 µg/mL Pen-Strep, 10 µg/mL of blasticidin, 100 µg/mL of Zeocin, and 1 µg/mL of Puromycin). The cell plates were dosed with 5 µL of the hCCR2-targeting-ADC samples, and then incubated at 37 °C for 20 hours. At the end of the incubation, 10 µL/well of the QUANTI-Luc™ (InvivoGen # rep-qlc1) were added, and luminescence was measured immediately using the LeadSeeker.

For the assay method described above, percent luminescence signal induction for each test ADC, at various concentrations, was calculated relative to untreated and control treated samples. Compound concentration versus percent signal induction curves were fitted to generate EC50 values.

**Pharmacokinetics evaluation in naïve mice**

For in vivo evaluation of the ADCs in naïve Balb/C mouse, female Balb/C mice at 6-8 weeks of age (purchased from Jackson Laboratory) were used. Mice were fed with normal diet and housed in a SPF animal facility in accordance with the Guide for Care and Use of Laboratory Animals and regulations of the Institutional Animal Care and Use Committee. Animals were kept at a temperature of 18-26 °C, a relative humidity of 50 ± 20% and intermittent light and dark cycles of 12 hours with food and water available ad libitum.

Pharmacokinetics of the ADCs were studied following injection of ADCs into Balb/C mice. Serum samples were taken at various time points and stored frozen for analysis.

The mouse plasma levels of total antibodies and conjugated payloads were measured by a 2-in-1 immunocapture based LC/MS assay on a Shimadzu UHPLC system interfaced to a Sciex 6500 QTRAP mass spectrometer. Briefly, mouse plasma samples were incubated with anti-human IgG coated magnetic beads for 45 min at room temperature, then non-specifically bound proteins were removed by washing the magnetic beads with PBST (PBS buffer at pH 7.4, containing 0.05% tween 20) and PBS buffer consecutively. After that, both naked antibodies (DAR=0) and ADCs (DAR ≥ 1) were eluted from the magnetic beads into 0.1% trifluoroacetic acid. After neutralizing the eluents and spiking in stable isotope labeled internal standards, one aliquot of sample was pipetted out and digested with papain for 1 hour at 37°C then used for the LC/MS analysis of conjugated payloads. The remaining samples were subjected to trypsin/lys-C digestion for 1 hour at 70°C then used for the LC/MS analysis of total antibodies.

The free payload in the circulation was also measured by LC/MS after performing plasma protein precipitation. In short, mouse plasma was mixed with 8 volumes of methanol containing stable isotope

labeled internal standard, then the supernatants were evaporated to dryness at 40°C under a gentle nitrogen stream. Finally, the residues were reconstituted in LC/MS grade water prior to LC/MS analysis.

### Assessment of mTAK-500 Pharmacokinetics in Tumor Bearing Mice

Female C57BL/6 mice, aged 7 to 8 weeks, were inoculated subcutaneous (SC) with 1.0× 10 MC38 tumor cells in the right flank. When tumors grew to approximately 300 to 500 mm3, animals were assigned into groups (n = 3/time point). Each group of animals received a single mTAK-500 administration via IV dosing as listed in Table 1. Animals were sacrificed at defined time points (Table 1), and tumor and plasma samples were harvested according to approved research operating procedures.

For plasma collection, approximately 500 µL of whole blood was obtained via cardiac puncture, placed into tubes coated with dipotassium ethylenediaminetetraacetic acid (K2EDTA) to prevent clotting, and centrifuged at 10,000 rpm for 5 minutes. Approximately 200 µL of plasma was then transferred into 1.4-mL sterile tubes, snap frozen on dry ice, and stored frozen at approximately −80°C. Tumor samples were excised from the mouse and placed into 1.4-mL sterile tubes snap frozen on dry ice and stored frozen at approximately −80°C for pharmacokinetic (PK) analysis. Samples were shipped on dry ice to Frontage Laboratories, Inc. (Exton, PA, USA), where they were stored in a freezer set to maintain −70°C ± 10°C until analysis.

Analysis of plasma and tumor samples was performed by personnel at Frontage Laboratories, Inc. following qualified LC/MS/MS methods: BTM-3181-R0 for total antibody and conjugated dazostinag from mTAK-500 in mouse K2EDTA plasma; BTM-3180-R0 for deconjugated dazostinag mouse K2EDTA plasma; BTM-3183-R0 for total antibody and conjugated dazostinag from mTAK-500 in mouse tumor homogenate; BTM-3182-R0 for deconjugated dazostinag in mouse tumor homogenate [15].

The lower limits of quantitation (LLOQ) for the assays were 30.0, 0.510, and 0.500 ng/mL for TAb, conjugated dazostinag and deconjugated dazostinag in plasma samples and 240, 4.08, and 4.00 ng/mL for TAb, conjugated dazostinag and deconjugated dazostinag in tumor samples, respectively.

Bioanalytical results were stored in Watson LIMS [version 7.6 (Thermo Systems [Philadelphia, PA, USA])] and were reported to 3 significant figures. TAb, conjugated dazostinag, and deconjugated dazostinag concentration results below the quantitation limit (BQL) for the respective analytes were reported in Watson as BQL and were treated as zero for statistical calculations.

The plasma tumor concentrations of TAb, conjugated dazostinag, and deconjugated dazostinag reported by Frontage Laboratories, Inc. [15] were used for the PK analyses and are reported in the text, figures, and tables. All dosing are shown in payload concentration unless mentioned otherwise. PK parameters were estimated using Phoenix WinNonlin [version 7.0 (Certara USA [Princeton, NJ, USA])] with a non-compartmental approach consistent with the intravenous (IV) bolus route of administration for TAb and conjugated dazostinag and extravascular route for deconjugated dazostinag. The conjugated dazostinag equivalent dose was calculated based on Equation 1.

**Equation 1**

Conjugated-payload equivalent dose = (ADC Dose/ADC MW) * drug antibody ratio (DAR) * payload MW

Where:

mTAK-500 MW: 156,000 g/mol.

DAZOSTINAG MW: 710.52 g/mol.

DAR: 3.0.

The area under the concentration-time curve (AUC) was calculated using the linear up log down method. The AUC was not reported for PK profiles with less than 3 quantifiable concentrations at consecutive time post dose. Concentrations of analytes that were BQL were set to 0.00 ng/mL for the purpose of calculating mean concentrations and the PK parameters. No statistical analyses were performed on the plasma and tumor concentration data or derived PK parameters. All derived parameters and associated standard deviations are reported to 3 significant figures, with the exception of time to reach Cmax (tmax), which is reported according to the PK sampling time with no decimal. Parameters relying on the determination of the terminal elimination phase were not reported if the coefficient of determination was less than 0.800, or if the percentage of the area under the curve extrapolated to infinity observed from time of the last observation to infinity represented more than 20% of the total area. AUC0-24h was used for exposure comparison between different dosing groups.

**In Vivo Assessment of mTAK-500 Efficacy in Tumor Bearing Mouse Models**

Seven-week-old female C57BL/6 mice were inoculated subcutaneously with 1.0× 106 MC38 tumor cells in the right flank. Tumor growth and body weights were monitored two times per week using vernier calipers and the mean tumor volume (MTV) was calculated using the formula [0.5× (length ×width2)]. When the MTV reached approximately 80 mm3, animals were randomized into treatment groups (n = 8/group) and dosed IV with either vehicle (PBS), mTAK-500 at 5 µg/kg, 10 µg/kg or 25 µg/kg as a single dose (days 0), or mKTI DAZOSTINAG at 5 µg/kg, 10 µg/kg or 25 µg/kg as a single dose (days 0).

Growth rate inhibition was calculated on day 13 and tumor volume was monitored through day 23 of the study to determine the number of complete responses, defined as a decrease in tumor volume to an undetectable size (<25 mm3). The mean maximum body weight loss was determined for each group using the mean body weight data from the treatment period, and the mean maximum percent body weight change was calculated on the basis of pre-dose body weights.

**Table S5**. In vitro STING activation in CCR2 overexpressing THP-1 cells

| | EC50 (nM) | Emax (%) |
|---|---|---|
| **TAK-500** | 1.8 | 92 |
| **ADC3** | 0.45 | 110 |
| **dazostinag** | 530 | 94 |
| **10** | 700 | 95 |

**Table S6**. In vitro plasma stability of ADC3 and TAK-500

| ADC | Payload loss (%) in human plasma | | | | Payload loss (%) in NHP plasma | | | | Payload loss (%) in mouse plasma | | | |
|---|---|---|---|---|---|---|---|---|---|---|---|---|
| | 1d | 2d | 3d | 4d | 1d | 2d | 3d | 4d | 1d | 2d | 3d | 4d |
| **ADC3** | 1.8 | 3.4 | 4.9 | 5.9 | 1.4 | 3.0 | 3.9 | 5.5 | 4.5 | 6.8 | 16.0 | 19.4 |
| **TAK-500** | 0.7 | 1.1 | 1.6 | 2.2 | 0.6 | 0.8 | 1.4 | 1.7 | 2.7 | 4.1 | 11.1 | 9.9 |

**Table S7**. Pharmacokinetic parameters of total Ab and conjugated payload in mouse and NHP after IV administration of TAK-500

| **Animal** | **Antibody dose (mg/kg)** | **Analyte** | **$T_{1/2}$ (hr)** | **$C_{1hr}$ (nM)** | **$AUC_{all}$ (hr*nM)** | **$AUC_{INF}$ (hr*nM)** | **CL (mL/hr/kg)** | **$V_{ss}$ (mL/kg)** |
|---|---|---|---|---|---|---|---|---|
| Mouse | 3 | Total Ab | 59 | 461 | 19100 | 21800 | 0.87 | 67 |
| | | Conj. payload | 36 | 1590 | 52300 | 54100 | 1.3 | 60 |
| NHP | 1 | Total Ab | 52 | 224 | 4090 | 4100 | 1.7 | 63 |
| | | Conj. payload | 17 | 857 | 9650 | 9400 | 2.9 | 45 |
| NHP | 3 | Total Ab | 40 | 657 | 14500 | 14400 | 1.4 | 49 |
| | | Conj. payload | 25 | 2510 | 33300 | 32900 | 2.4 | 53 |

**Table S8**. Comparison of pharmacokinetic parameters of total Ab and conjugated payload in naive mice after IV administration of isolated DAR species from TAK-500 (50 µg/kg, payload dose)

| **Reagent** | **Analyte** | **$T_{1/2}$ (hr)** | **$C_{1hr}$ (nM)** | **$AUC_{all}$ (hr*nM)** | **$AUC_{INF}$ (hr*nM)** | **CL (mL/hr/kg)** | **$V_{ss}$ (mL/kg)** |
|---|---|---|---|---|---|---|---|
| **TAK-500** | Total Ab | 59 | 461 | 19100 | 21800 | 0.87 | 67 |
| | Conj. payload | 36 | 1590 | 52300 | 54100 | 1.3 | 60 |
| **TAK-500, isolated DAR2** | Total Ab | 29 | 993 | 41350 | 41820 | 1.12 | 51 |
| | Conj. payload | 21 | 1424 | 44290 | 44410 | 1.59 | 57 |
| **TAK-500, isolated DAR4** | Total Ab | 75 | 408 | 21900 | 29880 | 0.65 | 76 |
| | Conj. payload | 44 | 1527 | 52260 | 56380 | 1.25 | 66 |
| **TAK-500, isolated DAR6** | Total Ab | 56 | 258 | 9750 | 10950 | 1.26 | 87 |
| | Conj. payload | 36 | 1329 | 38660 | 39940 | 1.76 | 74 |

| | | | | | | | |
|---|---|---|---|---|---|---|---|
| **TAK-500, isolated DAR8** | Total Ab | 45 | 117 | 4100 | 4400 | 2.2 | 103 |
| | Conj. payload | 32 | 920 | 28500 | 29100 | 2.6 | 85 |

**Table S9**. Pharmacokinetic parameters of total Ab, conjugated, and free payload in C57BL/6 mice bearing MC38 after IV administration of mTAK-500 at 2, 10, and 50 μg/kg (payload based)

| Dose | Matrix | Analyte | $T_{1/2}$ (hr) | $C_{1hr}$ (nM) | $AUC_{all}$ (hr*nM) | $AUC_{INF}$ (hr*nM) | CL (mL/hr/kg) | $V_{ss}$ (mL/kg) |
|---|---|---|---|---|---|---|---|---|
| 0.14 mg/kg (antibody) 2 μg/kg (payload) | Plasma | Total Ab | NC | 8.29 | 127 | NC | NC | NC |
| | | Conj. payload | NC | 26.9 | 396 | NC | NC | NC |
| 0.72 mg/kg (antibody) 10 μg/kg (payload) | Plasma | Total Ab | NC | 68.9 | 2569 | NC | NC | NC |
| | | Conj. payload | 32 | 226 | 6830 | 8655 | 1.63 | 75 |
| | Tumor | Total Ab | | 6.42 | 351 | | | |
| | | Conj. payload | | 15.4 | 913 | | | |
| | | Free payload | | 4.09 | 170 | | | |
| 3.59 mg/kg (antibody) 50 μg/kg (payload) | Plasma | Total Ab | 71 | 461 | 27529 | 33610 | 0.70 | 67 |
| | | Conj. payload | 47 | 1471 | 53878 | 58819 | 1.20 | 70 |
| | Tumor | Total Ab | | 58.1 | 8270 | | | |
| | | Conj. payload | | 123 | 18020 | | | |
| | | Free payload | | 8.54 | 663 | | | |

NC: not calculated due to the $\%AUC_{extrapolated}$ (percentage of the AUC extrapolated to infinity observed from time of the last observation [$t_{last}$] to infinity) represented more than 20% of the total area

## Spectral data

Compound **5**

1H NMR

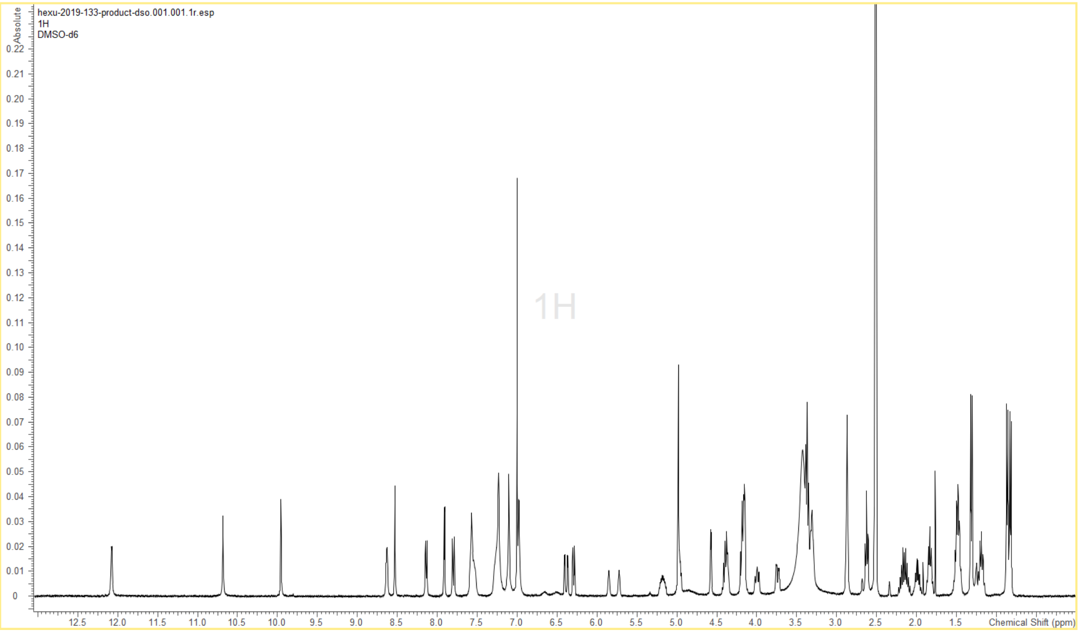


Compound **9**

1H NMR

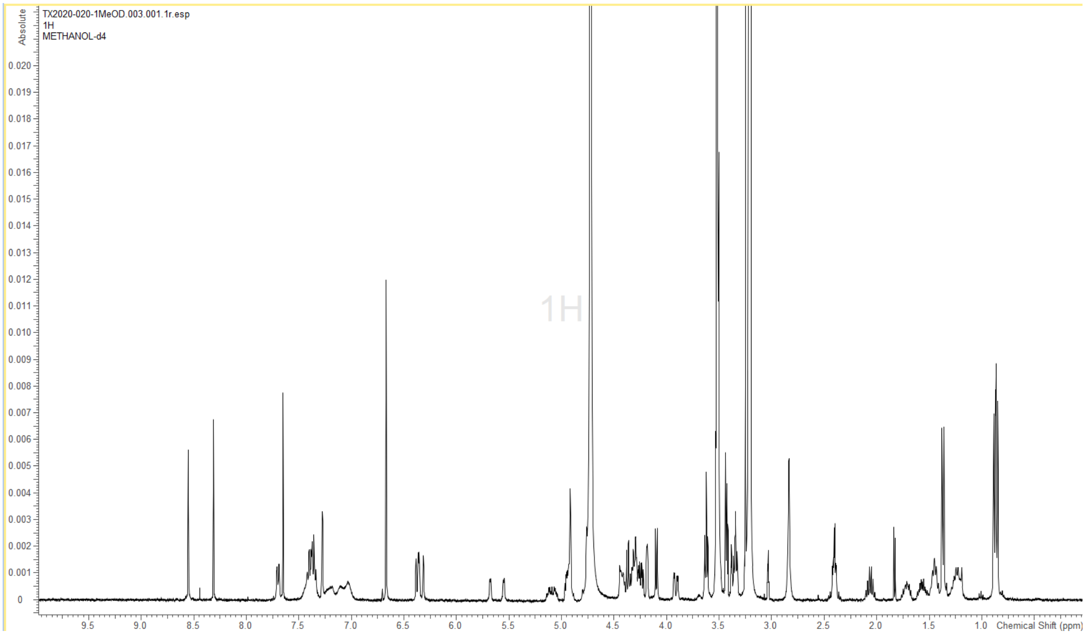

Compound **11**

1H NMR

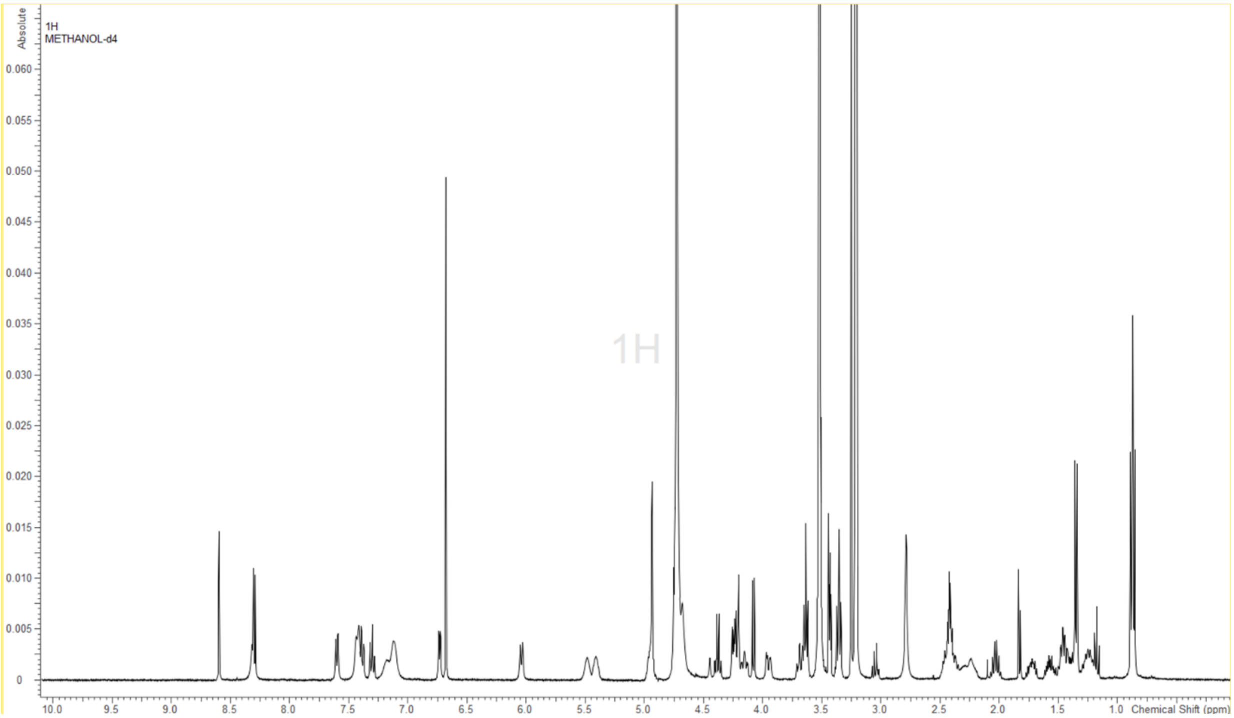


Compound **12**

1H NMR

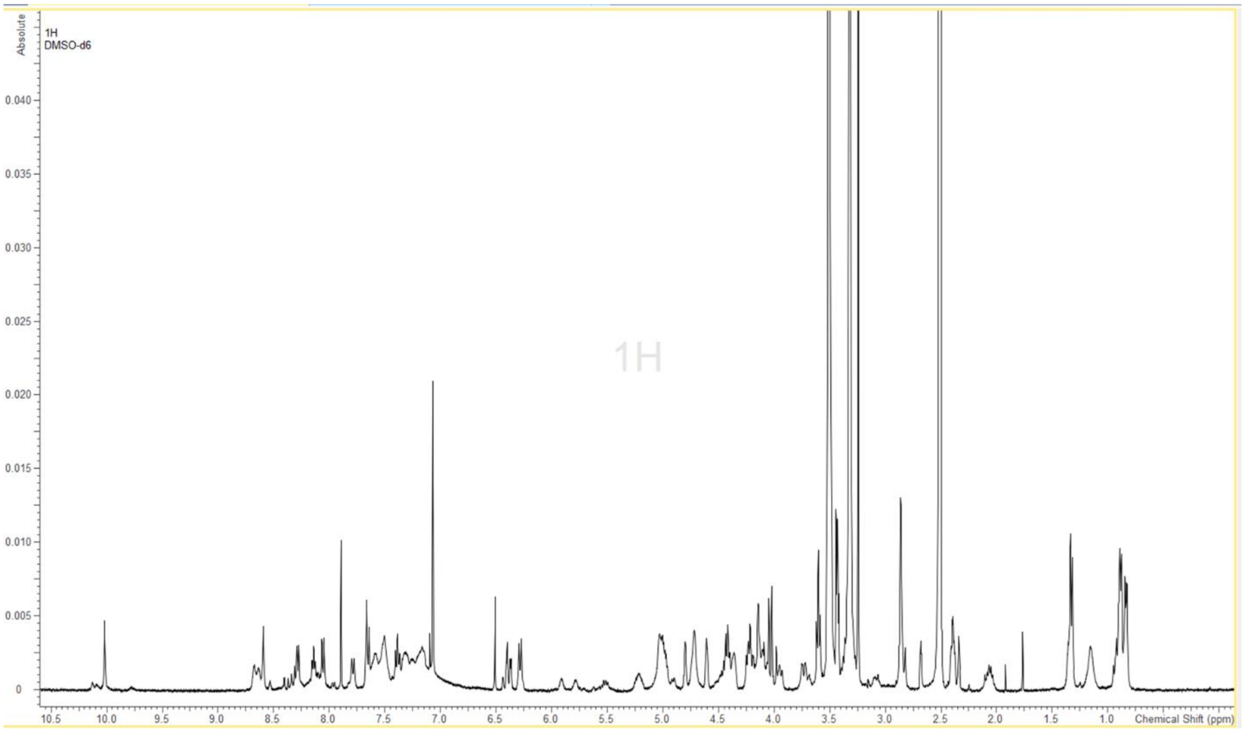

**TAK-500**

HIC

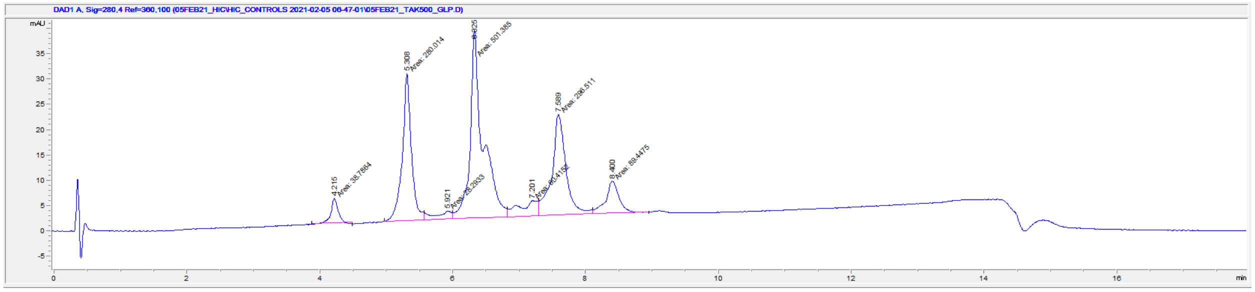

SEC

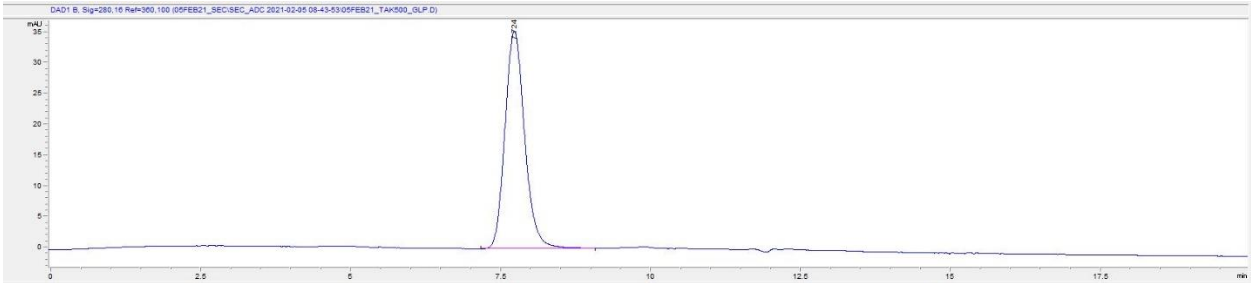

MS (heavy chain)

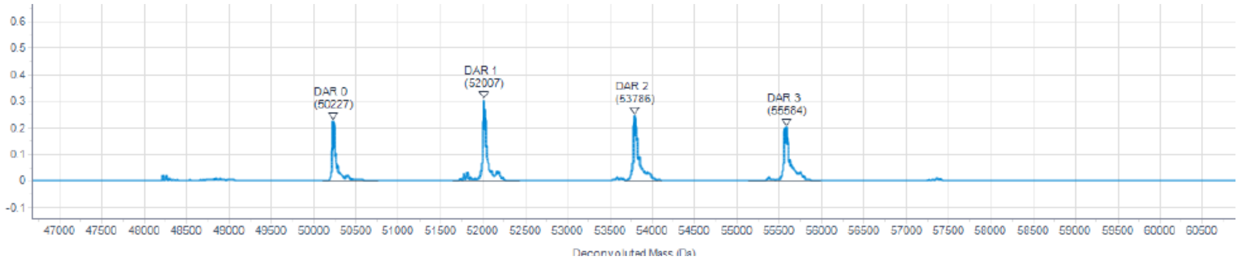


MS (light chain)

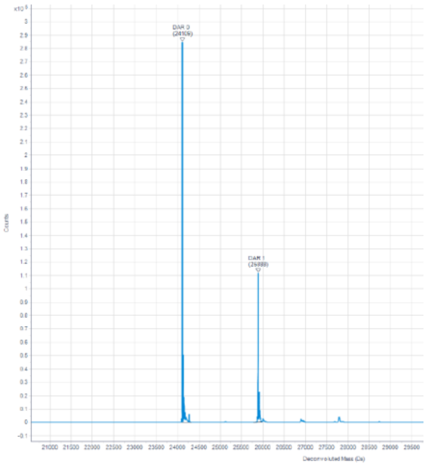

**ADC3**

HIC

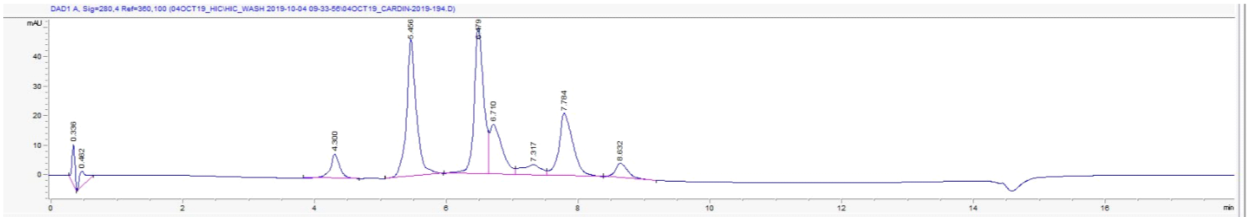


SEC

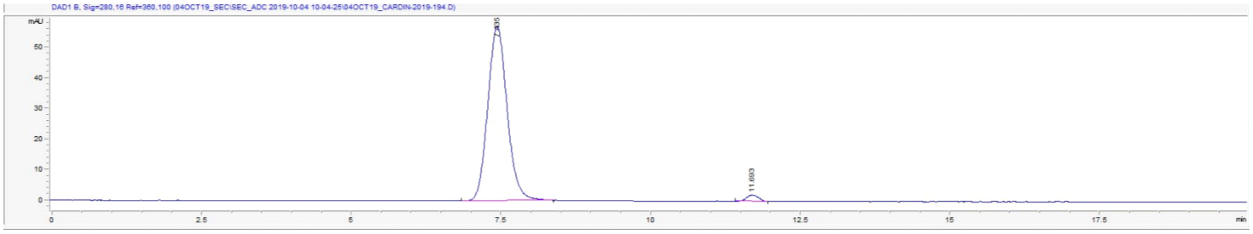


MS (heavy chain)

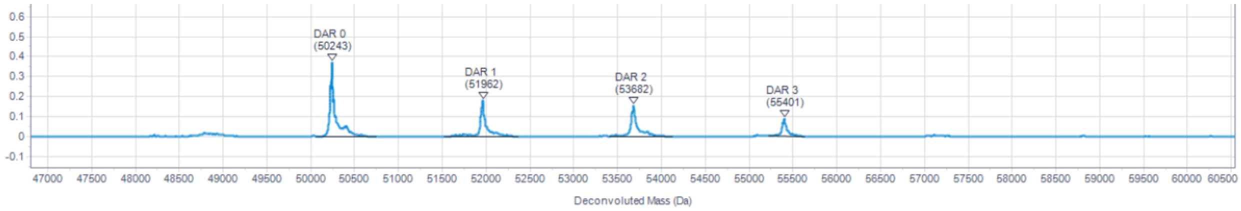


MS (light chain)

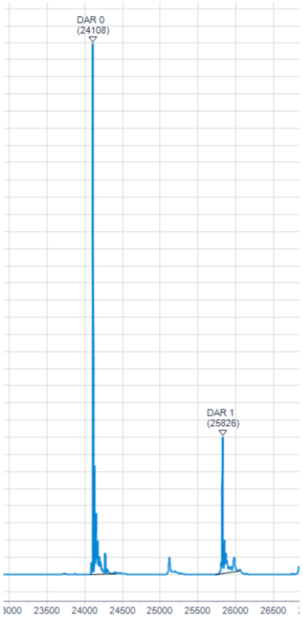

**ADC4**

HIC

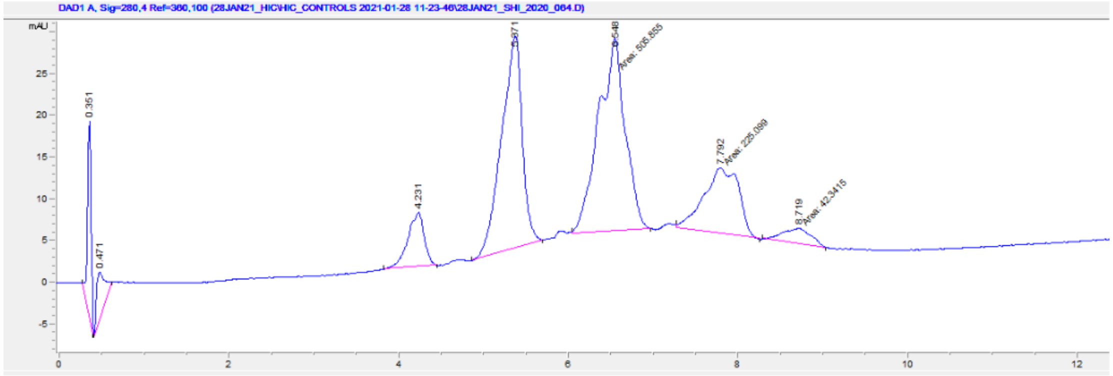


MS (before hydrolysis, heavy chain)

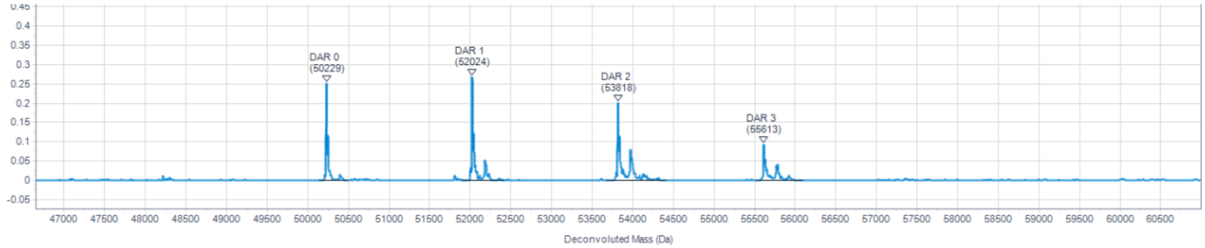


MS (after hydrolysis, heavy chain)

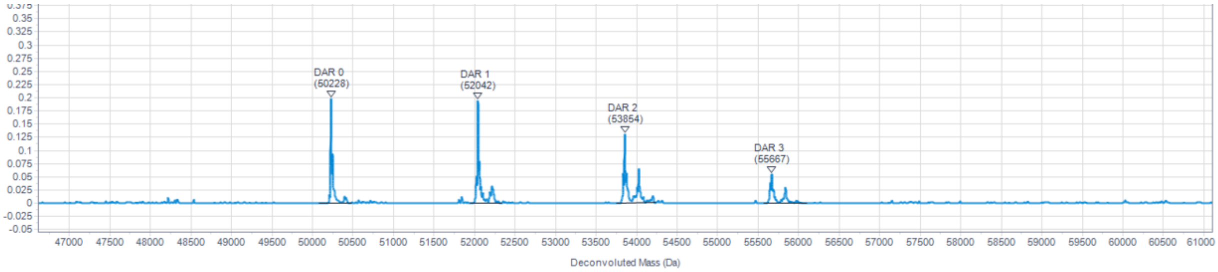


MS (after hydrolysis, light chain)

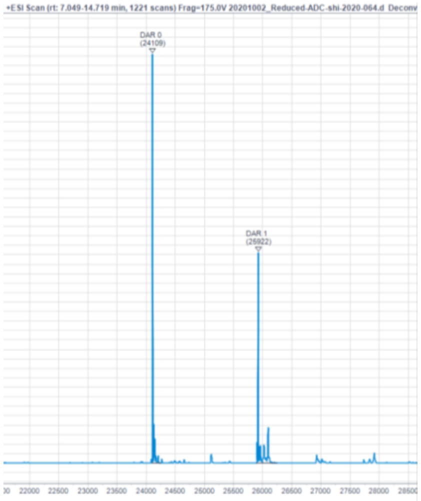


**mTAK-500**

HIC

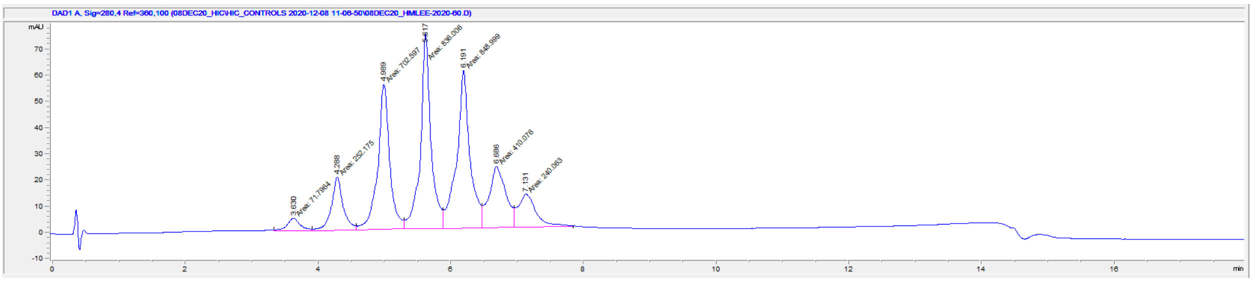


SEC

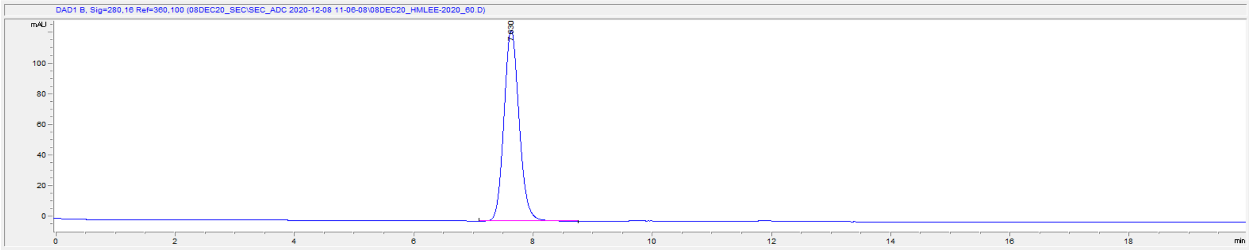


MS (heavy chain)

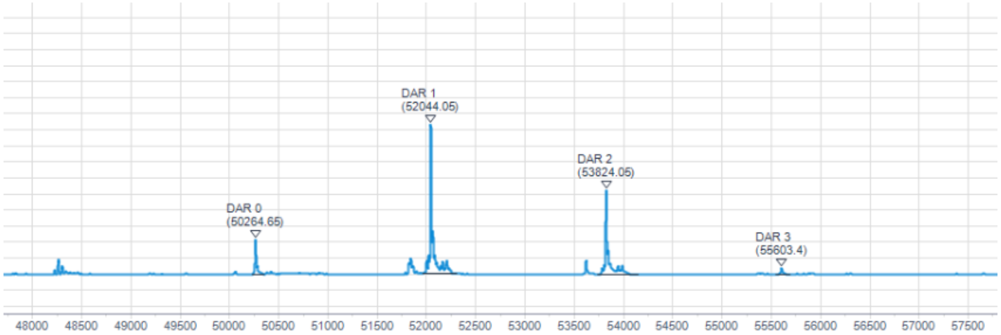

MS (light chain)

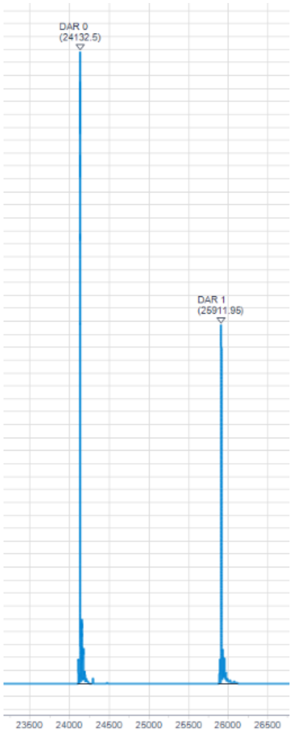

Supplement: Supplementary file 1 [file bc5c00424_si_001.pdf]
